# YAP1 controls the N-cadherin-mediated tumor-stroma interaction in melanoma progression

**DOI:** 10.1038/s41388-024-02953-1

**Published:** 2024-02-02

**Authors:** Yao Xiao, Linli Zhou, Thomas Andl, Yuhang Zhang

**Affiliations:** 1 Division of Pharmaceutical Sciences, College of Pharmacy, University of Cincinnati, Cincinnati, OH 45267 USA; 2 Burnett School of Biological Sciences, University of Central Florida, Orlando, FL 32816 USA

**Keywords:** Cancer microenvironment, Cell signalling

## Abstract

The hallmark of epithelial-to-mesenchymal transition (EMT) is the switch from epithelial cadherin (E-cadherin) to neural cadherin (N-cadherin), allowing melanoma cells to form a homotypic N-cadherin-mediated adhesion with stromal fibroblasts. However, how cadherin switching is initiated, maintained, and regulated in melanoma remains elusive. Here, we report a novel mechanism underlying cadherin switching in melanoma cells that is regulated by stromal Yes-associated protein 1 (YAP1) signaling. The progression of a BRAF-mutant mouse melanoma was suppressed in vivo upon YAP1 ablation in cancer-associated fibroblasts (CAFs). On the contrary, overexpressing YAP1 in CAFs accelerated melanoma development. By RNA-Seq, N-cadherin was identified as a major downstream effector of YAP1 signaling in CAFs. YAP1 silencing reduced N-cadherin expression in CAFs, leading to the downregulation of N-cadherin in neighboring melanoma cells. N-cadherin ablation inhibited the PI3K-AKT signaling pathway in melanoma cells and melanoma cell proliferation. The findings suggest that YAP1 depletion in CAFs induces the downregulation of p-AKT signaling in melanoma cells through the N-cadherin-mediated interaction between melanoma cells and CAFs. The data underscore an important role of CAFs in regulating N-cadherin-mediated adhesion and signaling in melanoma and highlight that disentangling cadherin-mediated cell-cell interactions can potentially disrupt tumor-stroma interactions and reverse the tumor cell invasive phenotype.

## Introduction

YAP1 is an oncoprotein and a downstream transcriptional coactivator in the Hippo signaling pathway [1]. YAP1 regulation by the highly conserved Hippo signaling pathway involves a cascade of protein serine kinases, including mammalian sterile twenty-like kinases 1/2 (MST1/2) and large tumor-suppressor kinases 1/2 (LATS1/2) [2]. When Hippo signaling is active, YAP1 is phosphorylated by LATS1/2 for cytoplasmic retention, ubiquitination, and degradation. When Hippo signaling is off, YAP1 remains unphosphorylated, translocates to the nucleus, and drives the transcription of target genes. The Hippo signaling pathway is considered a critical tumor-suppressor pathway, and its dysregulation has been noted in a variety of human cancers, in which YAP1 nuclear translocation abnormally activates target gene expression and enables cancerous cells to overcome contact inhibition, grow, and spread uncontrollably [3].

YAP1 activity is believed to be important for fibroblast activation, proliferation, and differentiation [4–6]. Lee et al. reported that YAP1 expression is highly increased in dermal fibroblasts in cutaneous wounds, and YAP1 silencing significantly delayed the wound-healing process [7]. CAFs in solid tumors have characteristics similar to myofibroblasts [8]. Calvo et al. reported that nuclear YAP1 activity is required to maintain CAF tumor-promoting phenotypes, including matrix stiffening, cancer cell invasion, and angiogenesis [9]. It is believed that reciprocal interactions between YAP1 activity in CAFs and tissue stiffness and mechanical stress in the tumor microenvironment drive a continuous process of maintaining CAF phenotypes and malignancy [10].

Previously, we showed that YAP1 is highly expressed in the nuclei of CAFs in human melanoma, and the biological functions of stromal fibroblasts, especially their extracellular matrix (ECM)-remodeling ability, are inhibited when YAP1 expression is silenced [6]. However, molecular mechanisms underlying the functional connection between YAP1 activity and the tumor-promoting functions of CAFs have not been fully identified. In this study, we established two mouse models that carry either YAP1-deficient CAFs or YAP1-overexpressing CAFs and demonstrated that YAP1 is important for CAFs to promote in vivo melanoma progression. YAP1-deficient CAFs exhibit significant cytoskeletal changes with reduced levels of actin stress fibers, focal adhesions, and myosins. We identified N-cadherin as a YAP1 downstream effector in CAFs. N-cadherin is a major molecule that mediates strong cell-cell adhesion and promotes cell viability and movement. Surprisingly, we found that N-cadherin reduction in CAFs induced the downregulation of N-cadherin in melanoma cells, subsequently causing inhibited p-AKT signaling and tumor cell proliferation. Taken together, our work uncovers a novel YAP1-regulated cellular mechanism that controls melanoma cell phenotypes and proliferation through the N-cadherin-mediated interaction between melanoma cells and CAFs.

## Results

### Stromal YAP1 signaling contributes to melanoma progression in vivo

**Fig. 1:**
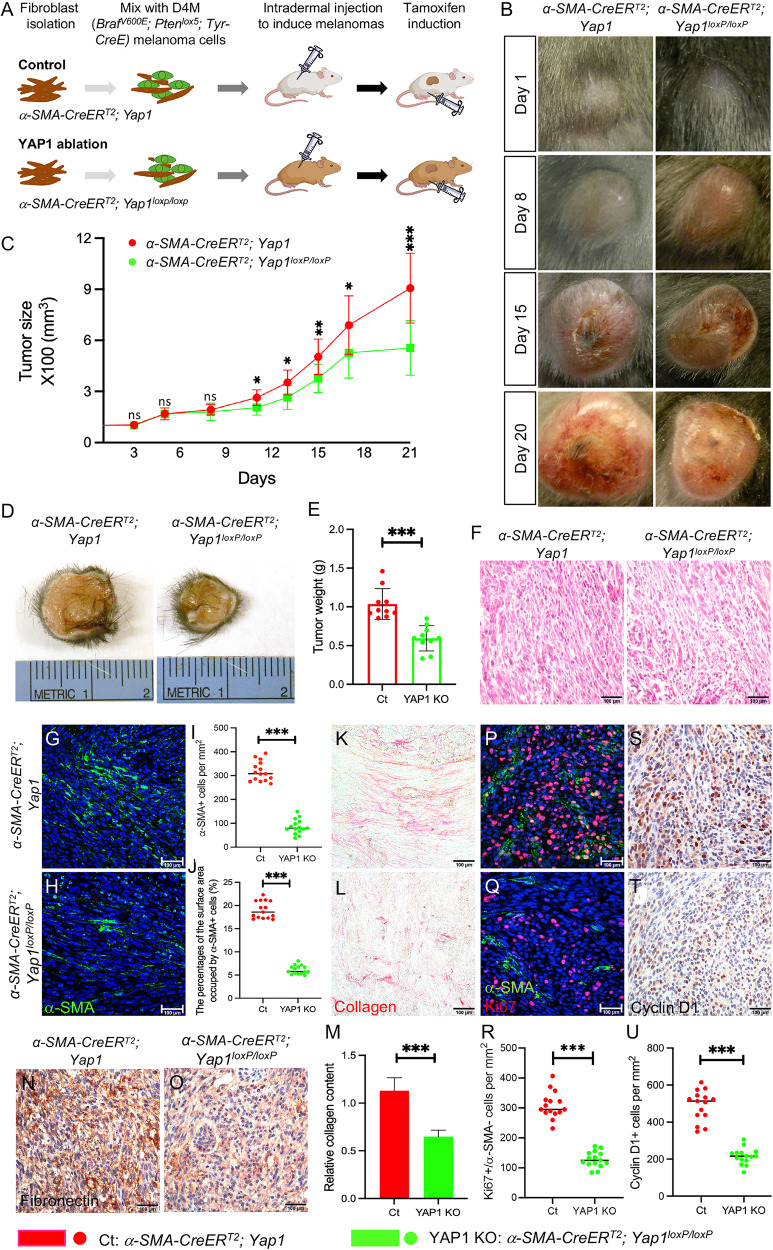
*Braf*^*V600E*^*; Pten*^*lox5/lox5*^ melanoma growth is suppressed by YAP1 deficiency in CAFs. **A** Illustration of the mouse model of melanoma for studying the in vivo effects of YAP1 ablation in CAFs on melanoma progression. D4M melanoma cells (green) mixed with uninduced control fibroblasts (*α-SMA-CreER*^*T2*^*; Yap1*) or mutant fibroblasts (*α-SMA-CreER*^*T2*^*; Yap1*^*loxp/loxp*^) were injected intradermally into the flanks of mice carrying the same genotypes as fibroblasts. Tumors were allowed to grow to a volume of approximately 62.5 cubic millimeters when tamoxifen was administered to induce YAP1 ablation in mutant *α-SMA-CreER*^*T2*^*; Yap1*^*loxp/loxp*^ CAFs and numbered day 1. The growth of tumors from control *Braf*^*V600E*^*; Pten*^*lox5/lox5*^-*α-SMA-CreER*^*T2*^*; Yap1* (Ct) and mutant *Braf*^*V600E*^*; Pten*^*lox5/lox5*^*-α-SMA-CreER*^*T2*^*; Yap1*^*loxp/loxp*^ (YAP1 KO) groups was monitored and compared. **B** Representative pictures of Ct melanoma (left panel) and YAP1 KO melanoma (right panel) on days 1, 8, 15, and 20. **C** Tumor sizes were measured and compared between Ct melanomas and YAP1 KO melanomas. *n* = 10. **D** Representative pictures of Ct and YAP1 KO melanomas on day 21. **E** Tumor weight comparison between Ct and YAP1 KO melanomas on day 21. Each data point represents the weight of one tumor on day 21. *n* = 10. The box indicates the average tumor weight, and error bars indicate the mean ± SD. **F** Histological sections of Ct melanoma and YAP1 KO melanoma were photographed under a light microscope. **G**, **H** Images show α-SMA staining of CAFs in Ct and YAP1 KO melanomas. The nuclei were counterstained using DAPI (blue). **I** The graph shows the numbers of α-SMA+ fibroblasts per mm^2^ counted in Ct and YAP1 KO melanomas. Fifteen random fields from three melanoma pairs were counted. *n* = 15. **J** The graph shows the percentages of the surface areas occupied by α-SMA+ fibroblasts measured in Ct and YAP1 KO melanomas. Fifteen random fields from three melanoma pairs were counted. *n* = 15. **K**, **L** Images show collagen staining of melanoma tissue sections in the indicated groups. **M** Quantitative comparison of collagen contents in Ct and YAP1 KO melanomas by collagen extraction and colorimetric measurement. *n* = 4. **N**–**O** Images show ECM protein fibronectin expression in Ct and YAP1 KO melanomas by immunohistochemistry. **P**, **Q** Images show α-SMA and Ki67 co-immunofluorescent staining of melanoma tissue sections as indicated. **R** Graph shows the comparison of the numbers of α-SMA- cells that are Ki67+ in each group. Each data point represents the number of α-SMA-; Ki67+ cells per mm^2^ counted in each melanoma. **S**, **T** Representative images of cyclin D1 staining of melanoma tissue sections in the indicated groups by immunohistochemistry. **U** Graph shows the numbers of cyclin D1+ cells in Ct and YAP1 KO melanomas. Each data point represents the number of cyclin D1+ cells per mm^2^ counted in one melanoma. For all staining pictures, the scale bar represents 100 µm. In all statistical graphs, data are represented as the mean ± SD. *P ≤ 0.05; **P ≤ 0.01; ***P ≤ 0.001; ns not significant.

To assess the contribution of YAP1 signaling in CAFs to BRAF-mutant melanoma progression in vivo, we generated a transgenic mouse model, *α-SMA-CreER*^*T2*^*; Yap1*^*loxP/loxP*^, which allows the inducible ablation of YAP1 expression in CAFs [11, 12]. In the *α-SMA-CreER*^*T2*^ transgene, the expression of sequestered Cre recombinase is driven by a α-smooth muscle actin (*α-SMA*) promoter [13], which is known to be a major gene expressed in CAFs [14]. *Yap1*^*loxP/loxP*^ harbors homozygous loxP-flanked *Yap1* alleles, which can be recombined into *Yap1*-null alleles by activated Cre recombinase [12]. To induce melanomas, isolated fibroblasts of genotype either *α-SMA-CreER*^*T2*^*; Yap1*^*loxP/loxP*^ (YAP1 group) or *α-SMA-CreER*^*T2*^*; Yap1* (control group) were mixed with oncogenic D4M melanoma cells, which carry the *Braf*^*V600E*^ activating mutation and are PTEN-deficient, and injected intradermally into the flanks of recipient mice of the same genotype as CAFs (Fig. 1A). When the tumors reached an approximate size of 62.5 mm^3^, the mice were administered tamoxifen by intraperitoneal injection to activate sequestered Cre recombinase and knock out the *Yap1* gene in *α-SMA-CreER*^*T2*^*; Yap1*^*loxP/loxP*^ CAFs (Fig. S1A, B), which are referred to as YAP1-deficient CAFs.

As shown in Fig. 1B, C, D4M melanomas carrying YAP1-deficient CAFs (named YAP1 KO) grew slower than tumors carrying CAFs expressing normal levels of YAP1 (named Ct). Twenty-one days after tamoxifen injection when mice were sacrificed, YAP1 KO melanomas were significantly smaller in size (Fig. 1D) and lighter in weight (Fig. 1E) (555.91 ± 160.70 mm^3^, 0.59 ± 0.16 g) than Ct tumors (906.43 ± 205.25 mm^3^, 1.04 ± 0.20 g), suggesting that D4M melanoma growth was suppressed upon the loss of YAP1 in CAFs. H&E staining revealed that the structure of YAP1 KO melanomas was less compact with more intercellular spaces than that of Ct tumors (Fig. 1F). The number of α-SMA-positive (α-SMA+) CAFs and the surface areas occupied by α-SMA+ cells were reduced in melanomas upon YAP1 ablation (Fig. 1G–J) albeit CAFs appeared not to be a dominant component of the tumor mass. CAFs are the major producers of collagen and ECM proteins in the tumor stroma. As shown in Fig. 1K–O, the levels of collagen fibers and fibronectin were significantly reduced in YAP1 KO melanomas. Consistent with the reduced YAP1 KO tumor size and weight, the number of melanoma cells (α-SMA-negative, α-SMA-) that were Ki67-positive (Ki67+) and the number of cyclin D1-positive (cyclin D1+) cells were both decreased in YAP1 KO melanomas (Fig. 1P–U). The data suggest that YAP1 activity is important for CAFs to support melanoma cell proliferation in vivo.

### YAP1 overexpression in CAFs accelerates tumor progression

**Fig. 2:**
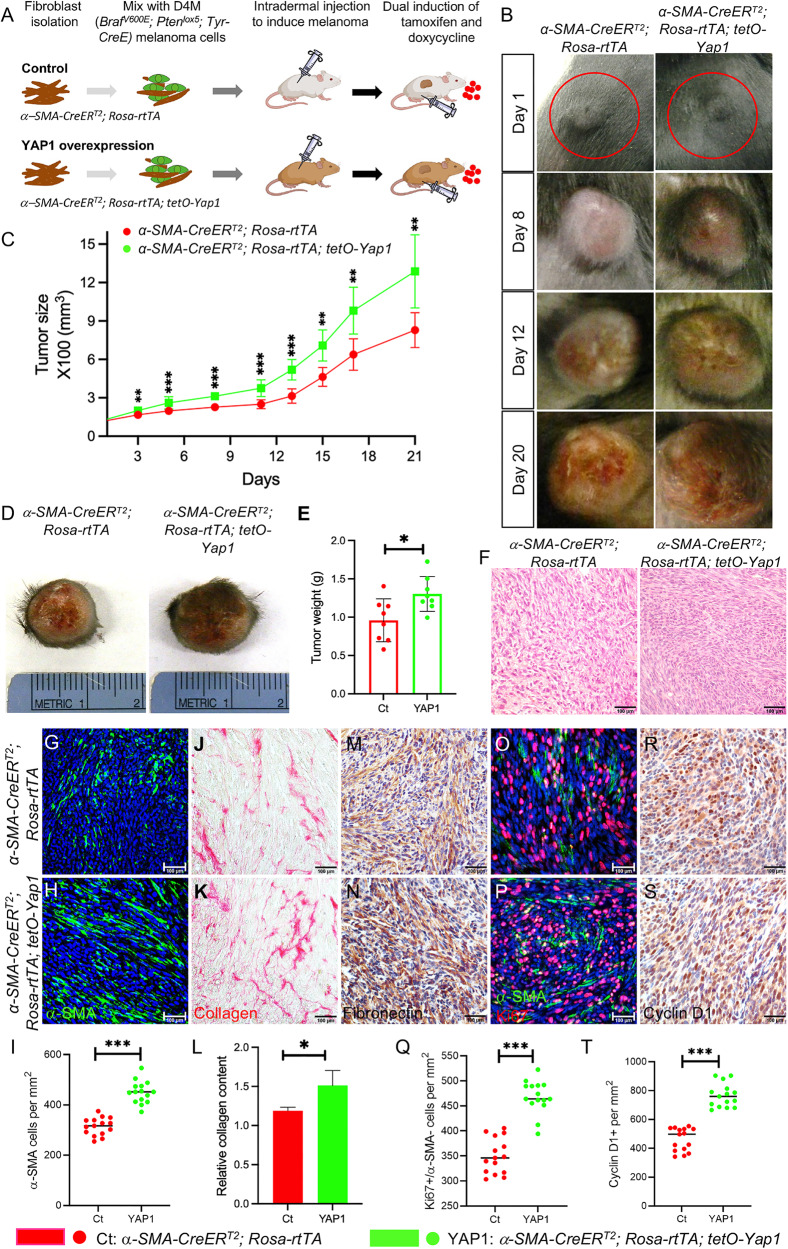
YAP1 overexpression in CAFs accelerates *Braf*^*V600E*^*; Pten*^*lox5/lox5*^ melanoma progression. **A** Illustration of the mouse model of melanoma for studying the in vivo effects of YAP1 overexpression in CAFs on melanoma progression. D4M melanoma cells (green) were mixed with either uninduced control fibroblasts (*α-SMA-CreER*^*T2*^*; Rosa-rtTA*) or mutant fibroblasts (*α-SMA-CreER*^*T2*^*; Rosa-rtTA; tetO-Yap1*) and then injected intradermally into the flanks of mice carrying the same genotypes as fibroblasts. Tumors were allowed to grow to a volume of approximately 62.5 cubic millimeters before tamoxifen was administered and mice were fed with a Dox diet (small red dots) to induce YAP1 overexpression in mutant CAFs. The growth of tumors from both control *Braf*^*V600E*^*; Pten*^*lox5/lox5*^*-α-SMA-CreER*^*T2*^*; Rosa-rtTA* (Ct) and mutant *Braf*^*V600E*^*; Pten*^*lox5/lox5*^*-α-SMA-CreER*^*T2*^*; Rosa-rtTA; tetO-Yap1* groups (YAP1) was closely monitored and compared. **B** Representative pictures of Ct melanomas and YAP1 melanomas on days 1, 8, 12, and 20. **C** Tumor size was measured and compared between Ct melanomas and YAP1 melanomas. *n* = 8. **D** Representative pictures of Ct melanoma and YAP1 melanoma on day 21. **E** Tumor weight comparison between the Ct and YAP1 groups. The graph shows the tumor weight comparison between the two groups on day 21. Each data point represents the weight of one tumor on day 21. *n* = 8. The box indicates the average tumor weight, and error bars indicate the mean ± SD. **F** Histological sections of Ct melanoma and YAP1 melanoma were photographed under a light microscope. **G**, **H** Images show α-SMA staining of CAFs in Ct and YAP1 melanomas. The nuclei were counterstained blue using DAPI. **I** Graph shows the numbers of α-SMA+ fibroblasts per mm^2^ counted in Ct and YAP1 melanomas. *n* = 15. **J**, **K** Images show collagen staining of melanoma tissue sections in the indicated groups (red). **L** Quantitative comparison of collagen contents in Ct and YAP1 melanomas by collagen extraction and colorimetric measurement. *n* = 3. **M**, **N** Images show ECM protein fibronectin expression in Ct and YAP1 melanomas by immunohistochemistry. **O**, **P** Images show α-SMA and Ki67 co-immunofluorescent staining of melanoma tissue sections in the indicated groups. **Q** Graph shows the numbers of α-SMA- melanoma cells that are Ki67+ in each group. Each data point represents the number of α-SMA-; Ki67^+^ cells per mm^2^ counted in each melanoma. **R**, **S** Representative images of cyclin D1 staining of melanoma tissue sections in the indicated groups by immunohistochemistry. **T** Graph shows the numbers of cyclin D+ cells in Ct and YAP1 melanomas. Each data point represents the number of cyclin D+ cells per mm^2^ counted in each melanoma. For all staining pictures, the scale bar represents 100 µm. In all statistical graphs, data are represented as the mean ± SD. *P ≤ 0.05; **P ≤ 0.01; ***P ≤ 0.001; ns not significant.

Next, we addressed the impact of upregulating YAP1 signaling in CAFs on melanoma progression. As shown in Fig. 2A, we generated a triple transgenic mouse strain, *α-SMA-CreER*^*T2*^; *Rosa-rtTA; tetO-Yap1*, that inducibly overexpresses YAP1 protein in α-SMA+ fibroblasts and determined whether YAP1-overexpressing α-SMA+ fibroblasts could accelerate melanoma progression. In the *tetO-Yap1* transgene, the expression of YAP1 is controlled by a doxycycline-inducible *tetO* promoter [15] and requires the presence of reverse tet transactivator (rtTA) and doxycycline. The expression of rtTA by the *Rosa-rtTA* transgene is prevented by a loxP-flanked STOP cassette [16]. Thus, the generation of rtTA can only occur when active Cre recombinase is present to remove the STOP cassette, which is provided by the *α-SMA-CreER*^*T2*^ transgene upon tamoxifen administration, leading to the overexpression of YAP1 protein in α-SMA+ CAFs (Fig. S1C, D), which are referred to as YAP1-overexpressing CAFs.

Interestingly, we observed that melanomas carrying YAP1-overexpressing CAFs (named YAP1) grew more quickly than control tumors carrying wild-type CAFs (Fig. 2B, C). On day 21, when the tumors were collected, the average size and weight of YAP1 melanomas (1287.99 ± 286.13 mm^3^, 1.30 ± 0.23 g) exceeded those of control tumors carrying normal CAFs (829.98 ± 136.48 mm^3^, 0.96 ± 0.28 g) (Fig. 2D, E). Histological analysis showed that YAP1 melanomas had a more compact internal structure than control tumors (Fig. 2F). The number of α-SMA+ CAFs per mm^2^ was increased from 314 ± 35 in control melanomas to 450 ± 44 in YAP1 melanomas (Fig. 2G-I). The contents of collagen (Fig. 2J–L) and fibronectin (Fig. 2M, N) were also higher in YAP1 melanomas than those in control melanomas. In contrast to the YAP1 KO mouse model, as shown in Fig. 2O–Q, overexpressing YAP1 in CAFs increased the number of Ki67+ melanoma cells per mm^2^ to 468 ± 33 compared to an average of 350 ± 35 in control melanomas. Similarly, the number of cyclin D1+ cells was also increased in YAP1 melanomas (Fig. 2R–T). The data further confirm that YAP1 indeed plays a crucial role in CAFs to support melanoma development.

### YAP1 regulates the biological properties of CAFs

**Fig. 3:**
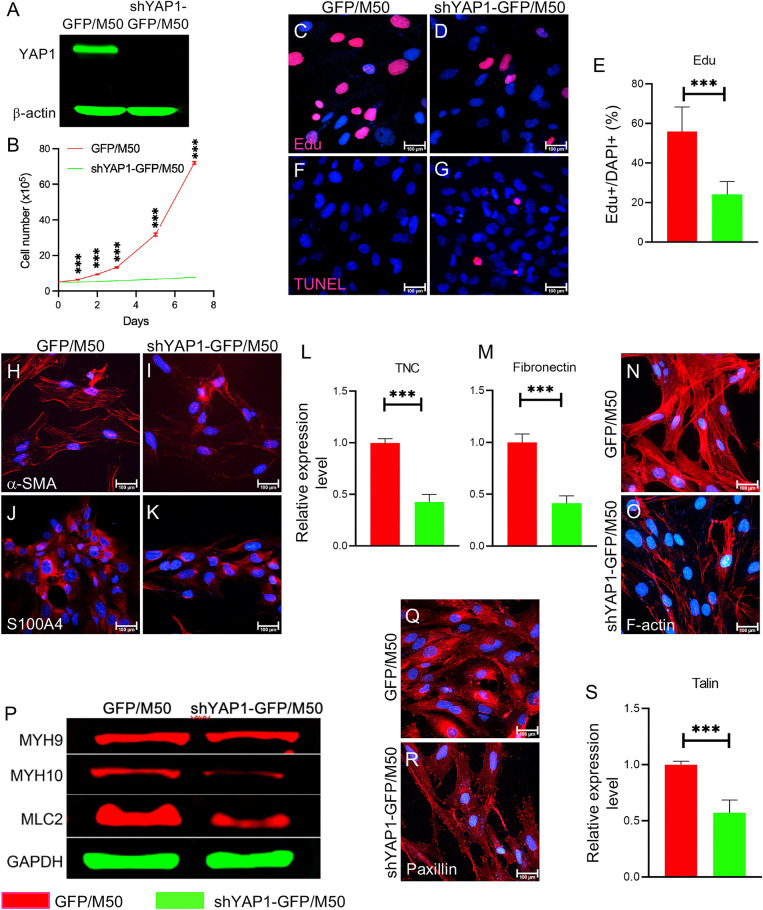
YAP1 is essential for the biological properties of CAFs. YAP1 in M50 was ablated using YAP1-GFP/Fb-3 shRNA (V3SH7669-226435710) and named as shYAP1-GFP/M50. The cells were treated with 500 ng/ml doxycycline for 72 h for indicated experiments unless otherwise stated. **A** Western blot shows YAP1 expression in cultured GFP/M50 and shYAP1-GFP/M50 cells. **B** The numbers of GFP/M50 and shYAP1-GFP/M50 in the culture medium were counted at days 0, 1, 2, 3, 5, and 7 for comparison. *n* = 3. **C**, **D** Images show EdU staining of GFP/M50 and shYAP1-GFP/M50 with blue DAPI nuclear counterstaining. **E** Graph shows the statistical comparison of the percentages of EdU+ cells in cultured GFP/M50 and shYAP1-GFP/M50 cells from three independent experiments. **F**, **G** Images show TUNEL+ cells in cultured GFP/M50 and shYAP1-GFP/M50 cells by TUNEL assay. **H**–**K** Images show α-SMA and S100A4 staining of GFP/M50 and shYAP1-GFP/M50. **L**, **M**. The expression of the ECM proteins TNC and fibronectin in GFP/M50 or shYAP1-GFP/M50 cells was quantified by qPCR. GAPDH was used as an internal control. *n* = 3 **N**, **O** Images show F-actin staining of GFP/M50 and shYAP1-GFP/M50 with DAPI counterstaining. **P** Western blot showing the expression of MYH9, MYH10, and MLC2 in GFP/M50 and shYAP1-GFP/M50 cells. **Q**, **R** Images show paxillin staining of GFP/M50 and shYAP1-GFP/M50 with DAPI counterstaining. **S** The expression of talin in GFP/M50 and shYAP1-GFP/M50 cells was quantified by qPCR. GAPDH was used as an internal control. *n* = 3. The expression levels of the indicated proteins were normalized to the expression level of GAPDH. For all staining pictures, the scale bar represents 100 µm. In all statistical graphs, data are represented as the mean ± SD. *P ≤ 0.05; **P ≤ 0.01; ***P ≤ 0.001; ns not significant.

To evaluate the mechanism that controls CAF phenotypes by YAP1 signaling, we established inducible YAP1-deficient CAFs using shRNA in different human melanoma-derived CAF cell lines, including M27 and M50 [17]. Three different YAP1-targeting shRNAs (Fig. S2A–D) were evaluated for their efficiencies in silencing YAP1 expression. YAP1-GFP/Fb-3 shRNA (V3SH7669-226435710) was found to have the highest inhibitory efficiency and was selected for subsequent experiments (Fig. S2E, F). CAFs transduced with V3SH7669-226435710 were named shYAP1-GFP/M50 and shYAP1-GFP/M27. Nontargeting shRNA-transduced M27 and M50 were used as the controls and named GFP/M50 and GFP/M27, respectively. YAP1 depletion in M27 and M50 was confirmed by Western blotting and qPCR (Fig. 3A and S2G, H). The loss of YAP1 expression suppressed the proliferation of M50 and M27 cells (Fig. 3B and S3A) but did not cause increased cell apoptosis, which was confirmed by EdU staining and TUNEL assay (Fig. 3C–G and S3B, C). The expression of CAF markers, including α-SMA and S100A4 [14], was strongly decreased in YAP1-deficient CAFs (Fig. 3H–K and S3F–I). Quantification analysis showed reduced fibronectin and Tenascin C (TNC) contents in YAP1-deficient CAFs (Fig. 3L–M and Fig. S3D–E).

CAFs exhibit remodeled cytoskeletal structure with increased intracellular tension. As shown in Fig. 3N–P and S3J–O, YAP1 deficiency led to reduced levels of stress fiber F-actin, myosin heavy chain MYH10, and myosin light chain MLC2 in CAFs. In addition, the expression of the focal adhesion proteins paxillin and talin, which are involved in cytoskeletal regulation, was suppressed (Fig. 3Q–S and S3P, Q). The data suggest that YAP1 function is involved in the control of the cytoskeletal machinery. To confirm that the observed phenotypes are indeed caused by YAP1 deficiency, we used another shRNA, YAP1-GFP/Fb-1 shRNA (V3SH7669-225152043), to silence YAP1 expression in M50 (named shYAP1-GFP/M50-1). As shown in Fig. S4A–Q, the data demonstrated that the suppressed biological properties of CAFs are due to YAP1 ablation and not caused by the off-target effects of the shRNA.

To exclude the possibility that the inhibitory effects of stromal YAP1 deficiency on melanoma progression shown in Fig. 1 were contributed by YAP1 loss in other α-SMA-expressing cell types in recipient mice, we established a human melanoma xenograft model using a combination of GFP/M50 or shYAP1-GFP/M50 with A375 melanoma cells (Fig. S5A). In this xenograft model, YAP1 ablation only occurs in shYAP1-GFP/M50. As shown in Fig. S5B–D, on day 21, the size and weight of melanomas formed by A375 and shYAP1-GFP/M50 tumors were significantly smaller and lighter, respectively, than those consisting of A375 and GFP/M50. The number of α-SMA+ CAFs and contents of collagen and fibronectin were both significantly reduced in melanomas upon YAP1 ablation (Fig. S5E–J). As expected, the number of melanoma cells (α-SMA-) that were Ki67+ was decreased in melanomas carrying YAP1-deficient CAFs (Fig. S5K, L).

### CAFs require YAP1 to maintain matrix remodeling ability

**Fig. 4:**
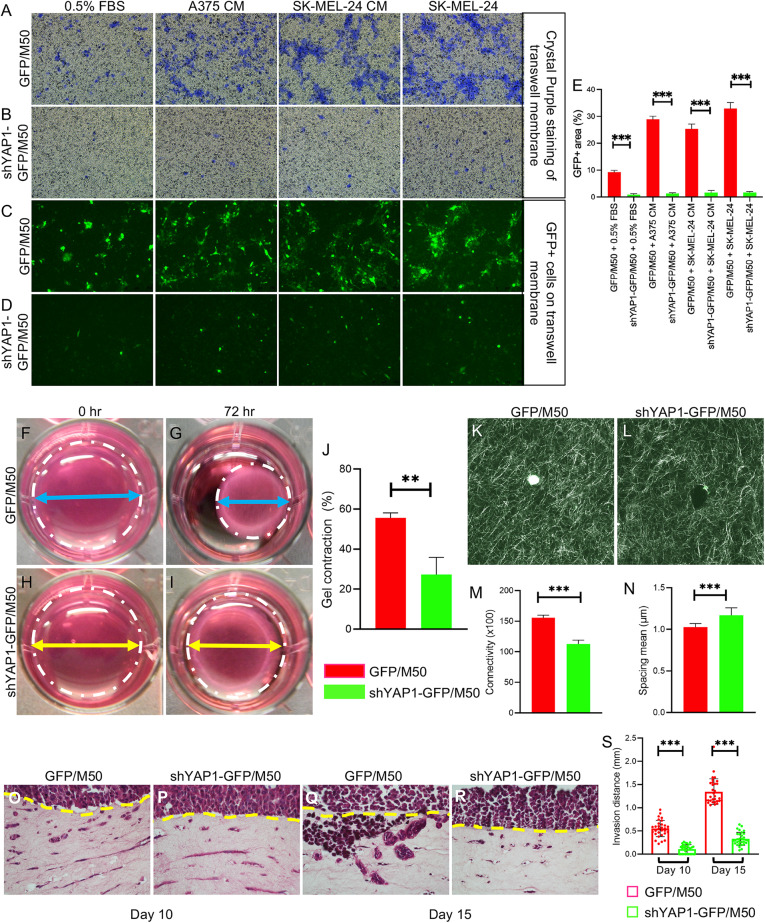
YAP1 regulates the migratory and ECM remodeling abilities of CAFs. The cells were treated with 500 ng/ml doxycycline for 72 h for indicated experiments unless otherwise stated. Images show the migratory response of GFP/M50 and shYAP1-GFP/M50 to the indicated culture conditions at 48 h in the transwell migration assay by crystal violet staining (**A**, **B**) and green fluorescence imaging (**C**, **D**). **E** Bar graph shows the percentages of the area occupied by green fluorescent GFP/M50 and shYAP1-GFP/M50 on the Transwell membranes at the 48th hour. *n* = 6. **F**–**I** Representative images of collagen gel contraction assays using GFP/M50 and shYAP1-GFP/M50 at 0 and 72 h. The gel in each well is circled by a white dashed line. The blue (GFP/M50) and yellow (shYAP1-GFP/M50) arrow lines indicate the diameter of the contracted gels. **J** Statistical quantification of the relative percentages of gel contraction by GFP/M50 and shYAP1-GFP/M50 from 0 to 72 h. *n* = 3. **K**–**L** CRM images of gels embedded with GFP/M50 and shYAP1-GFP/M50 at 72 h. **M**, **N** CRM images were analyzed using ImageJ with the BoneJ plugin to compare connectivity and spacing between the gels embedded with GPF/M50 and shYAP1-GFP/M50. **O**–**R** Representative images of melanoma cell invasion in collagen gels embedded with GFP/M50 and shYAP1-GFP/M50 after 10 and 15 days. *n* = 3. The original front of the melanoma cells is indicated by a yellow line. **S** Statistical quantification of the migrating distances by melanoma cells in the gels embedded by GFP/M50 and shYAP1-GFP/M50 at day 10 and day 15. In all statistical graphs, data are quantified and shown as the mean ± SD. *P ≤ 0.05; **P ≤ 0.01; ***P ≤ 0.001; ns not significant.

Migration, matrix contraction, and ECM remodeling require cytoskeletal contraction and remodeling [18, 19]. Since YAP1 deficiency led to reduced expression of specific cytoskeletal proteins, we assessed the contribution of YAP1 to the migratory ability of CAFs using the transwell migration assay. As shown in Fig. 4A–E, after 48 hours, when cultured in DMEM with 0.5% FBS, the percentage of migrating GFP-positive (GFP+) shYAP1-GFP/M50 cells on the insert membrane was 0.92 ± 0.35%, which was significantly less than that of GFP/M50 (9.25 ± 0.71%). More significantly, the percentages of the GFP+ area on the membrane were increased to 28.54 ± 0.74% and 25.73 ± 1.19% when GFP/M50 was cultured in the media conditioned by A375 and SK-MEL-24 CM, respectively. The percentage increased to 33.26 ± 2.58% when SK-MEL-24 cells were seeded directly in the bottom well. However, shYAP1-GFP/M50 failed to respond to all stimulations. The results were confirmed using M27 (Fig. S3R) and the second shRNA in M50 (Fig. S4R).

Gel contraction assays showed that the ability of CAFs to remodel the collagen matrix was reduced after YAP1 depletion (Figs. 4F–J, S3S–V, and S4S–V). Next, to visualize and quantify collagen fiber alignment and reorganization, collagen fiber distribution was assessed using confocal reflection microscopy (CRM). The density of collagen fibers in the gel was significantly lower in collagen gel embedded with shYAP1-GFP/M50 than that in collagen gel embedded with GFP/M50 (Fig. 4K, L). CRM analysis revealed decreased connectivity and increased fiber spacing in collagen gel embedded with shYAP1-GFP/M50 due to YAP1 deficiency (Fig. 4M, N). The CRM data correlated well with the results shown by the collagen gel contraction assays, highlighting the importance of YAP1 for the ability of CAFs to remodel the ECM.

To understand how the ECM remodeled by CAFs influences the invasion of melanoma cells, we performed a 3D collagen gel invasion assay. GFP/M50 or shYAP1-GFP/M50 cells were embedded in collagen gel with melanoma cell A375 added on top of collagen gels. Histological staining showed a significant reduction in melanoma cell invasion in collagen gel populated with shYAP1-GFP/M50 (Fig. 4O–R). On day 15, the distance of melanoma cells invading in gel embedded with GFP/M50 was four times greater than that of shYA–P1-GFP/M50 (1.34 ± 0.28 mm vs. 0.33 ± 0.14 mm in Fig. 4S), suggesting that CAFs require YAP1 to make the gel accessible for A375 to invade.

### N-cadherin is a YAP1 target in CAFs

**Fig. 5:**
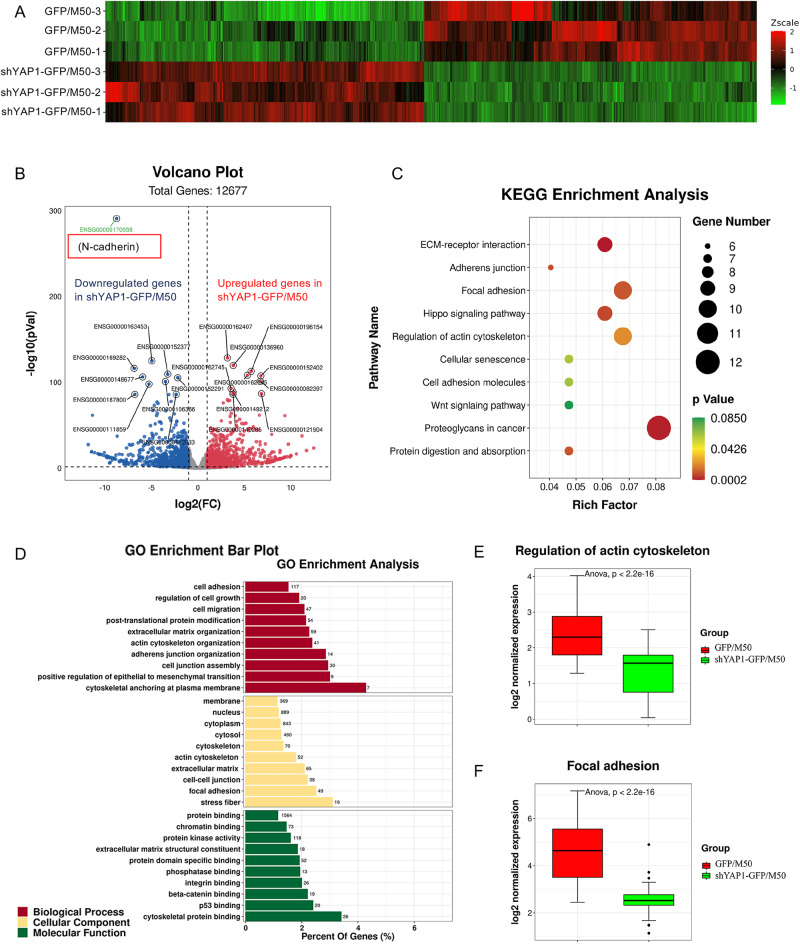
N-cadherin is a YAP1 target in CAFs. **A** Heatmap of the expression patterns (Z-scaled FPKM values) of the differentially expressed genes between GFP/M50 and shYAP1-GFP/M50. Changes in expression levels are displayed from green (less expressed) to red (more expressed). **B** Volcano plot shows the relationship between the fold-change (on the X-axis) and the significance of the differential expression test (Y-axis) for each gene between GFP/M50 and shYAP1-GFP/M50. Red dots represent upregulated genes and blue dots represent downregulated genes in shYAP1-GFP/M50 compared to GFP/M50. Gray dots represent the genes that are not significantly differentially expressed between GFP/M50 and shYAP1-GFP/M50. **C** The scatter plot shows the KEGG enrichment analysis results. The size and color of dots represent gene numbers and *p* values. The rich factor is the ratio of the number of differentially expressed genes enriched in the indicated pathway to the number of all genes annotated in this pathway. **D** The bar plot shows GO enrichment analysis results in three major GO subclasses for downregulated genes using the GO database. **E**, **F** Gene set enrichment analysis (GSEA) demonstrated that YAP1 has a significant correlation with biological functions related to regulation of the actin cytoskeleton and focal adhesion.

To obtain a global picture of the underlying mechanisms by which YAP1 regulates the functional properties of CAFs and identify YAP1-regulated genes that are involved in a CAF-elicited melanoma program, we performed RNA-Seq to compare the gene expression profiles between GFP/M50 and shYAP1-GFP/M50. As shown in Fig. 5A, a list of differentially expressed genes was generated by high-stringency and comparative analysis of RNA-Seq data using the Air platform (https://transcriptomics.sequentiabiotech.com/). A total of 1147 genes that were upregulated at least twofold and 1140 genes that were downregulated at least twofold in shYAP1-GFP/M50 are shown in a volcano plot (Fig. 5B). We were particularly interested in understanding the genes that were downregulated in shYAP1-GFP/M50, as they may be relevant for identifying the YAP1-mediated mechanism by which CAFs interact with melanoma cells. Therefore, KEGG pathway analysis and Gene Ontology (GO) enrichment analysis were performed [20–22]. As shown in Fig. 5C, KEGG enrichment analysis of the top 300 downregulated genes revealed that proteoglycans in cancer, regulation of actin cytoskeleton, and focal adhesion are the most affected pathways in CAFs after the loss of YAP1 expression. The most enriched subclasses by GO enrichment analysis of all 1140 downregulated genes were cytoskeletal anchoring at the plasma membrane, stress fiber, and cytoskeletal protein binding (Fig. 5D). Gene set enrichment analysis (GSEA) showed that the expression levels of the genes in the KEGG pathways of regulation of actin cytoskeleton and focal adhesion were indeed suppressed in shYAP1-GFP/M50 compared to those of GFP/M50 (Fig. 5E, F). Interestingly, among the downregulated genes, CDH2, the gene encoding N-cadherin, appears to be the most significantly downregulated gene in CAFs upon YAP1 ablation, as shown in the volcano plot (Fig. 5B), and could potentially function as a key downstream effector of YAP1 signaling in CAFs due to its known role in tumor cell-fibroblast adhesion.

### N-cadherin deficiency in CAFs leads to the downregulation of N-cadherin in melanoma cells

**Fig. 6:**
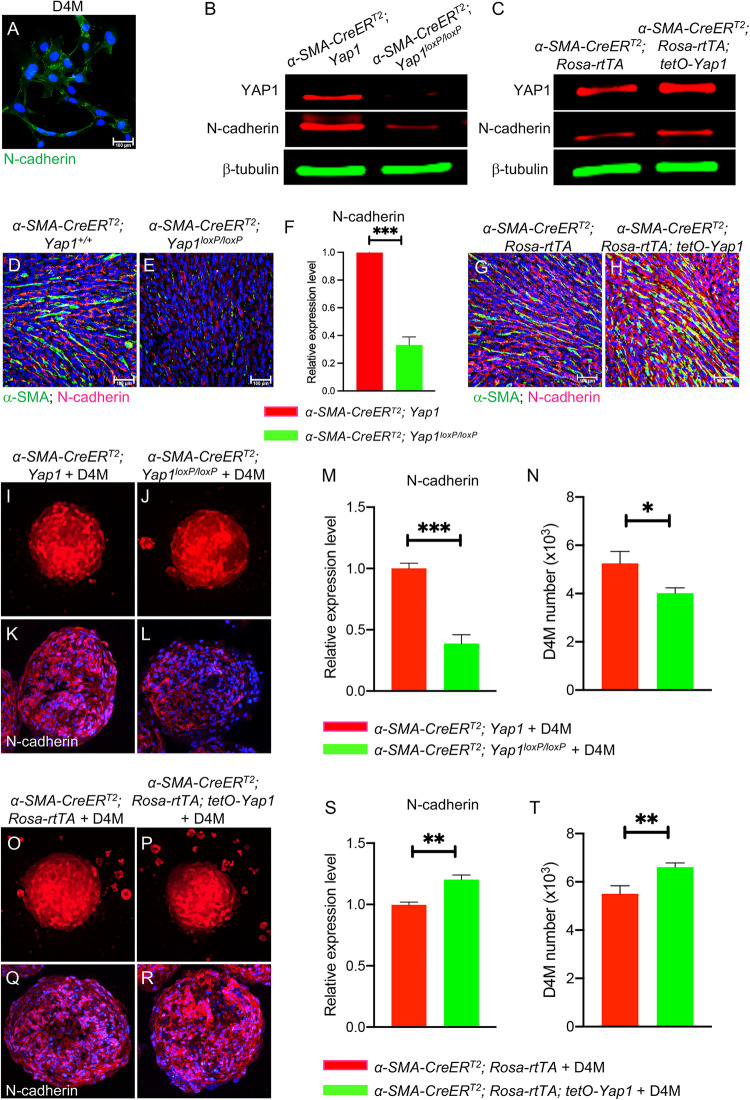
N-cadherin deficiency in fibroblasts leads to N-cadherin downregulation in co-cultured mouse melanoma cells. **A** Representative image shows N-cadherin staining of D4M mouse melanoma cells with blue DAPI nuclear counterstaining. **B** Western blot shows the loss of N-cadherin in fibroblasts upon YAP1 ablation. **C** Western blot shows the upregulated expression of N-cadherin in YAP1-overexpressing fibroblasts. **D**, **E** α-SMA and N-cadherin expression in mouse melanomas containing control fibroblasts (**B** ***α****-SMA-CreER*^*T2*^*; Yap1*) or YAP1-deficient fibroblasts (**C** *α-SMA-CreER*^*T2*^*; Yap1*^*loxp/loxp*^) was visualized by co-immunostaining using an anti-α-SMA antibody (green) and anti-N-cadherin antibody (red). **F** Quantification of N-cadherin expression in the indicated mouse melanoma tissues by qPCR and normalized to the internal control GAPDH. *n* = 3. **G**, **H** α-SMA and N-cadherin expression in mouse melanomas containing control fibroblasts (**D** *α-SMA-CreER*^*T2*^*; Rosa-rtTA*) or YAP1-overexpressing fibroblasts (**E** *α-SMA-CreER*^*T2*^*; Rosa-rtTA; tetO-Yap1*) was visualized by co-immunostaining using an anti-α-SMA antibody (green) and anti-N-cadherin antibody (red). **I**, **J** Fluorescence images show RFP-tagged D4M cells in co-cultured spheroids as indicated. **K**, **L** Fluorescence images show N-cadherin staining of co-cultured spheroids as indicated using an anti-N-cadherin antibody (red). **M** Quantitative analysis of N-cadherin expression in co-cultured spheroids as indicated by qPCR. *n* = 3. **N** Graph shows the numbers of D4M cells in co-cultured spheroids as indicated. *n* = 3. **O**, **P** Fluorescence images show RFP-tagged D4M cells in co-cultured spheroids as indicated. **Q**, **R** Fluorescence images show N-cadherin staining of co-cultured spheroids as indicated using an anti-N-cadherin antibody (red). **S**: Quantitative analysis of N-cadherin expression in co-cultured spheroids as indicated by qPCR. *n* = 3. **T** Graph shows the numbers of D4M cells in co-cultured spheroids as indicated. *n* = 3. For all staining pictures, the scale bar represents 100 µm. In all statistical graphs, data are represented as the mean ± SD. *P ≤ 0.05; **P ≤ 0.01; ***P ≤ 0.001; ns not significant.

N-cadherin is a transmembrane protein that is known to function in cell‒cell adhesion [23]. Importantly, the E-cadherin to N-cadherin switch is known as a major part of the EMT event in tumor progression and metastasis [24]. D4M mouse melanoma cells express N-cadherin in culture (Fig. 6A). However, N-cadherin expression is lost in YAP1-deficient fibroblasts (Fig. 6B) while the level of N-cadherin is increased in YAP1-overexpressing fibroblasts (Fig. 6C). We then checked the expression of N-cadherin in two mouse melanoma models formed by D4M and two types of fibroblasts (shown in Figs. 1 and 2). As shown in Fig. 6D–F, the expression of N-cadherin was markedly reduced, especially in melanoma cells (α-SMA-), in melanoma containing YAP1-deficient CAFs. In contrast, the expression of N-cadherin was increased in both CAFs and melanoma cells (Fig. 6H) when YAP1 was overexpressed in CAFs. The data clearly demonstrated that N-cadherin expression in melanoma cells is modulated by YAP1-regulated N-cadherin expression in CAFs. The findings were further confirmed by co-culturing D4M cells with either YAP1-deficient fibroblasts, YAP1-overexpressing fibroblasts, or control fibroblasts using an in vitro 3D coculture system for N-cadherin expression assessment (Fig. 6I–T). Particularly, D4M cell number was reduced in co-cultured spheroids formed by D4M with mouse YAP1-deficient fibroblasts (Fig. 6N), while D4M number was increased in co-cultured spheroids formed by D4M with mouse YAP1-overexpressing fibroblasts (Fig. 6T).

**Fig. 7:**
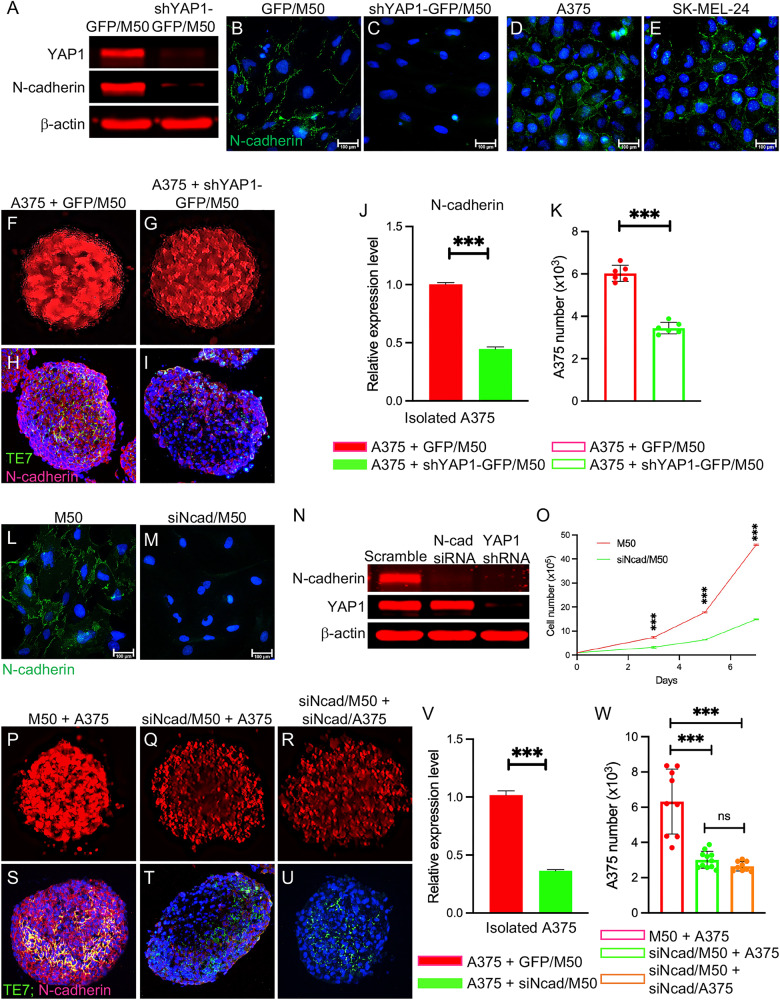
N-cadherin deficiency in CAFs leads to N-cadherin downregulation in melanoma cells. **A** Western blot shows the loss of N-cadherin in shYAP1-GFP/M50 cells upon YAP1 ablation. **B**–**E** Images show N-cadherin staining of GFP/M50, shYAP1-GFP/M50, A375 and SK-MEL-24 cells with blue DAPI nuclear counterstaining. **F**, **G** Fluorescence images show RFP-tagged A375 cells in co-cultured spheroids as indicated. **H**, **I** Images show TE7 and N-cadherin staining of co-cultured spheroids as indicated using an anti-TE7 antibody (green) and an anti-N-cadherin antibody (red). **J** Quantitative analysis of N-cadherin expression in A375 cells isolated from co-cultured spheroids as indicated by qPCR. *n* = 3. **K** Graph shows the numbers of A375 cells in co-cultured spheroids as indicated. *n* = 6. **L**, **M** Images show N-cadherin staining of scramble siRNA-transfected M50 and N-cadherin siRNA-transfected M50 (siNcad/M50) with blue DAPI nuclear counterstaining. **N** Western blot shows that N-cadherin expression was silenced in siNcad/M50 with normal YAP1 expression. In contrast, YAP1 ablation in M50 led to N-cadherin downregulation. Scrambled siRNA was used as a control. **O** Comparison of the numbers of siNcad/M50 and scramble siRNA-transfected M50 cells in culture for seven days. *n* = 3. **P**–**R** Fluorescence images show RFP-tagged A375 cells in co-cultured spheroids as indicated. **S**–**U** Images show TE7 and N-cadherin staining of co-cultured spheroids as indicated using an anti-TE7 antibody (green) and an anti-N-cadherin antibody (red). **V** Quantitative analysis of N-cadherin expression in A375 cells isolated from co-cultured spheroids as indicated. *n* = 3. **W** Graph shows the numbers of A375 cells in co-cultured spheroids as indicated. *n* = 9. For all staining pictures, the scale bar represents 100 µm. In all statistical graphs, data are represented as the mean ± SD. *P ≤ 0.05; **P ≤ 0.01; ***P ≤ 0.001; ns not significant.

Western blotting and immunostaining demonstrated the loss of N-cadherin in two human CAFs upon YAP1 silencing using two different shRNAs (Figs. 7A–C, S6A–C, and S7A–C). N-cadherin is expressed in the human melanoma cell lines A375 and SK-MEL-24 (Fig. 7D, E). We first checked the expression of N-cadherin in xenografts (Fig. S5), in which YAP1 was ablated in CAFs. As shown in S5M, N, the expression of N-cadherin was markedly reduced in α-SMA- melanoma cells. Next, we co-cultured A375 cells with either GFP/M50 or shYAP1-GFP/M50 cells and assessed N-cadherin expression. The spheroids formed by A375 and shYAP1-GFP/M50 appeared to have more interspaces and lower levels of N-cadherin expression (Fig. 7G, I) than the spheroids formed by A375 and GFP/M50 (Fig. 7F, H). The observation of a loose spheroid structure is consistent with what we observed in mouse melanoma carrying YAP1-deficient CAFs (Fig. 1F). Because A375 was tagged with red fluorescence, the number of A375 cells in each spheroid was counted and isolated. As expected, the spheroids formed by A375 and YAP1-deficient M50 contained a lower number of A375 than the ones formed by A375 and GFP/M50 (Fig. 7K), which also had lower N-cadherin expression (Fig. 7J). The findings were confirmed using YAP1-deficient M50 silenced using a different shRNA (YAP1-GFP/M50-1) (Fig. S6D–I) and YAP1-deficient M27 (Fig. S7D–I).

To confirm that ablating N-cadherin expression in CAFs does have similar effects as YAP1 depletion and to substantiate our finding that N-cadherin is the major downstream target of YAP1, we used siRNA to silence N-cadherin expression in GFP/M50 and GFP/M27 cells. After depleting N-cadherin expression, YAP1 was still expressed in CAFs (Fig. 7L–N). The loss of N-cadherin in M50 suppressed cell proliferation (Fig. 7O), partially mirroring the effects of YAP1 deficiency on CAF proliferation shown in Fig. 3B. We utilized an in vitro 3D coculture system to determine whether N-cadherin deficiency in CAFs can lead to a change in N-cadherin in melanoma cells. As shown in Figs. 7T, V, and S7M, N, depleting N-cadherin in CAFs led to the downregulation of N-cadherin in the melanoma cell lines A375 and SK-MEL-24. Spheroids were digested using collagenase to make single-cell suspensions for cell counting. The numbers of A375 and SK-MEL-24 cells in the spheroids were significantly reduced after the loss of N-cadherin expression in CAFs but close to the number of A375 cells in the spheroids formed by N-cadherin-deficient A375 and N-cadherin-deficient M50 cells (Figs. 7W and S7O). To confirm that the observed results are not caused by the off-target effects of a single siRNA, we used a different siRNA to silence N-cadherin expression in M50 and obtained similar data as shown in Fig. S8.

### N-cadherin loss in melanoma cells downregulates p-AKT signaling

**Fig. 8:**
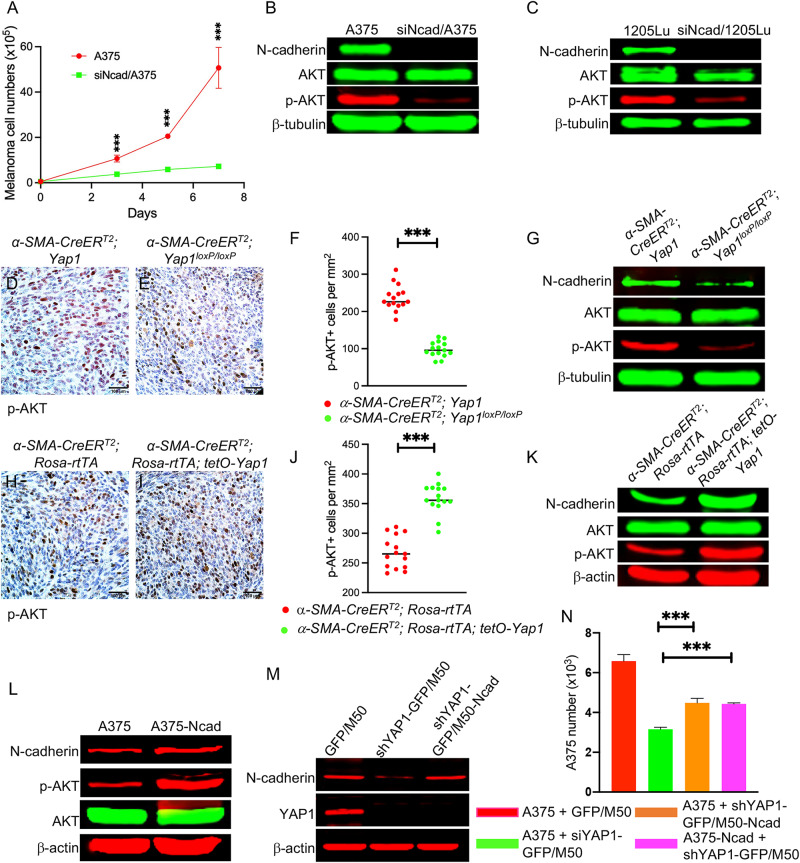
N-cadherin downregulation inhibits PI3K/AKT signaling in BRAF-mutant melanoma cells. **A** Comparison of the numbers of N-cadherin siRNA-transfected A375 (siNcad/A375) and scramble siRNA-transfected A375 cells in culture for 7 days. *n* = 3. **B** Western blot shows N-cadherin, AKT, and p-AKT expression in siNcad/A375 and A375 cells transfected with scramble siRNA. **C** Western blot shows N-cadherin, AKT, and p-AKT expression in 1205Lu cells transfected with scramble siRNA or N-cadherin siRNA (siNcad/1205Lu). **D**, **E** Representative images of p-AKT expression in melanomas containing control fibroblasts (*α-SMA-CreER*^*T2*^*; Yap1*) or YAP1-deficient fibroblasts (*α-SMA-CreER*^*T2*^*; Yap1*^*loxp/loxp*^). **F** Graph shows the numbers of p-AKT+ cells per mm^2^ counted in melanomas as indicated. *n* = 15. **G** Expression of N-cadherin, AKT and p-AKT in melanoma cells isolated from indicated melanomas was assessed by Western blotting. β-tubulin was used as an internal control. **H**, **I** Representative images of p-AKT expression in melanomas containing control fibroblasts (*α-SMA-CreER*^*T2*^*; Rosa-rtTA*) or YAP1-overexpressing fibroblasts (*α-SMA-CreER*^*T2*^*; Rosa-rtTA; tetO-Yap1*). **J** Graph shows the numbers of p-AKT+ cells per mm^2^ counted in indicated melanomas. *n* = 15. **K** Expression of N-cadherin, AKT and p-AKT in melanoma cells isolated from indicated melanomas was assessed by Western blotting. β-actin was used as an internal control. **L** Western blot shows N-cadherin, AKT, and p-AKT expression in A375 cells and N-cadherin-overexpressing A375 cells (A375-Ncad). **M** Western blot shows the expression of N-cadherin in YAP1-deficient M50 cells by lentiviral transduction (shYAP1-GFP/M50-Ncad). Normal M50 was used as a control. **N** Graph shows the numbers of A375 cells in co-cultured spheroids as indicated. *n* = 3. In all statistical graphs, data are represented as the mean ± SD. *P ≤ 0.05; **P ≤ 0.01; ***P ≤ 0.001; ns not significant.

It was reported previously that N-cadherin contributes to the proliferation of different cell types via the PI3K/AKT signaling pathway [25]. We examined the proliferation of melanoma cells after N-cadherin ablation and found that N-cadherin depletion in A375 and SK-MEL-24 indeed led to reduced cell proliferation (Figs. 8A and S7P). As shown in Figs. 8B, C and S7Q, p-AKT was significantly decreased in N-cadherin-deficient melanoma cell lines, including A375, 1205Lu, and SK-MEL-24, suggesting that N-cadherin in melanoma cells regulates the activation of the PI3K/AKT signaling pathway. Furthermore, the number of p-AKT-positive (p-AKT+) cells was significantly reduced when YAP1 expression was silenced in CAFs in the melanoma stroma (Fig. 8D–F). However, an increased number of p-AKT+ cells was found in the melanomas containing YAP1-overexpressing CAFs (Fig. 8H–J). To confirm that AKT signaling was indeed downregulated or upregulated in melanoma cells upon YAP1 ablation or overexpression in CAFs, tumor cells were isolated from melanomas for AKT Western blotting. As shown in Fig. 8G, K, both N-cadherin and p-AKT were reduced in melanoma cells in the tumors containing YAP1-deficient CAFs and upregulated in the tumors containing YAP1-overexpressing CAFs, suggesting that AKT signaling in BRAF-mutant melanoma cells was, at least partially, regulated through the N-cadherin-N-cadherin interaction between melanoma cells and CAFs.

Moreover, we overexpressed N-cadherin in YAP1-deficient CAFs (Fig. 8M) and melanoma cells (Fig. 8L) to understand if the phenotypes in melanoma cells caused by YAP1 deficiency in CAFs can be rescued by N-cadherin activity. As shown in Fig. 8L, overexpressing N-cadherin in A375 upregulated AKT signaling. By 3D spheroid culture, we found that the reduction in A375 numbers in the spheroids formed by shYAP1-GFP/M50 and A375 was partially rescued by either N-cadherin-overexpressing YAP1-deficient CAFs in spheroids formed with A375 or N-cadherin-expressing A375 in spheroids formed with YAP1-deficient M50. The data demonstrated that the N-cadherin-N-cadherin tumor-stroma interaction is indeed modulated by YAP1 signaling in CAFs, and the AKT signaling pathway is an N-cadherin downstream pathway in melanoma cells.

## Discussion

CAFs are known to have diverse functions, including ECM deposition and remodeling, growth factor secretion, and extensive signaling exchange with melanoma cells and other types of stromal cells [26]. One interesting and important characteristic of CAFs that has been described by Calvo et al. is the contribution of YAP1 as a master driver of the CAF phenotype [9]. We previously reported that YAP1 is a part of the β-catenin signaling axis in CAFs that controls CAF behaviors and demonstrated that loss of either of them is detrimental to CAF function [6, 17, 27]. In this study, two mouse models carrying either YAP1-deficient CAFs or YAP1-overexpressing CAFs showed opposite effects on in vivo melanoma growth, reflecting the importance of YAP1 in CAFs and of CAFs for melanoma development. In addition, the suppressive phenotypes contributed by YAP1 deficiency in CAFs were faithfully replicated in human melanoma xenografts. Nevertheless, the reduction in melanoma size and weight in the mouse melanoma model and human xenograft model is a combinatorial outcome of the reduced melanoma cell proliferation and loss of CAFs in tumor stroma. However, α-SMA+ cells did not appear to be a major component of the tumor mass. As such, the loss of CAFs is not considered to be a major factor responsible for the tumor weight loss caused by YAP1 ablation in CAFs.

RNA-Seq revealed that the gene that stands out and is most downregulated in CAFs after YAP1 loss is N-cadherin, a molecule that spearheads CAF-melanoma interactions and tumor invasion after melanoma cells undergo the switch from E-cadherin to N-cadherin [28]. N-cadherin is known to be expressed on CAFs and associated with the actin cytoskeleton through catenin molecules [29]. We confirmed the loss of N-cadherin in CAFs upon YAP1 ablation in both mouse fibroblasts and human CAFs. Furthermore, increased YAP1 expression in CAFs upregulated N-cadherin levels. Our results of YAP1 regulating N-cadherin have also been documented in other cell types. For example, suppressing YAP1 expression reduces N-cadherin expression in glioma cells [30]. In addition, YAP1 activity and N-cadherin expression can both be regulated by matrix stiffness [31].

The E- to N-cadherin switch in tumor cells is principally the switch from an invasion-suppressing cadherin [32] to a motility and invasion-promoting cadherin [33]. Melanocytes interact with keratinocytes and are retained in the epidermis since both express E-cadherin. When melanoma cells become invasive, the switch from E-cadherin to N-cadherin expression allows them to break away from keratinocytes, bind to N-cadherin-expressing fibroblasts, and invade the underlying dermis [34]. Forced expression of N-cadherin in N-cadherin-negative melanocytes conferred elevated properties of migration to these nonmalignant cells [35]. Accordingly, reducing N-cadherin expression in human melanoma cell lines leads to decreased invasive capabilities [36]. Different mechanisms that control the E- to N-cadherin switch in tumor cells have been reported [28], including the PI3K/PTEN pathway that transcriptionally regulates the ‘cadherin switch’ through Twist and Snail [37]. However, it is unclear whether CAFs have any role in regulating this switch and influencing melanoma progression through the N-cadherin interaction.

Interestingly, we observed that the loss of N-cadherin expression in CAFs reduces N-cadherin in melanoma cells and their proliferative phenotype. N-cadherin, E-cadherin, and other adhesion receptors are continuously turned over at the membrane and contribute to the dynamic assembly and disassembly of adhesive junctions [38–40]. Upon internalization in response to different signals, cadherin molecules are either recycled or degraded. However, it remains to be determined how the loss of N-cadherin in CAFs leads to the loss of N-cadherin in melanoma cells. One possible reason could be that the direct interaction between melanocytes and fibroblasts via N-cadherin is closely regulated. When CAFs lose N-cadherin molecules, homotypic N-cadherin interactions can no longer be maintained. Thus, melanoma cells initiate the degradation process to remove unneeded N-cadherin and downregulate N-cadherin expression. Another explanation could be that YAP1-deficient or N-cadherin-deficient CAFs are unable to produce cytokines or molecules that are necessary for the maintenance of N-cadherin expression and membrane presentation in melanoma cells. Potentially, ablating N-cadherin in CAFs could constitute a tumor inhibitory signal and reverse the invasive phenotypes in melanoma cells since cadherin-mediated cell-cell adhesion is central to bridging neighboring cells and the cytoskeleton for sensing and responding to physical and mechanical changes in the stroma.

How changes in N-cadherin expression bring about these profound changes in cellular behaviors in melanoma cells is partially understood. It has been demonstrated that N-cadherin may regulate PI3K-AKT signaling [35, 41]. In our study, we not only confirmed that N-cadherin downregulation in melanoma cells reduces the AKT signaling activity but also proved that expressing N-cadherin in YAP1-deficient CAFs and melanoma cells can at least, partially, rescue the inhibitory effects on melanoma cell proliferation and p-AKT signaling due to the loss of N-Cadherin-mediated intercellular interactions between BRAF-mutant melanoma cells and CAFs. This surprising finding highlights the importance of melanoma-stroma interactions and their largely untouched potential for novel therapeutic approaches [26].

Ablating or blocking N-cadherin in melanoma cells impairs their cell-cell adhesion, suppresses migration, and reduces their viability, suggesting that N-cadherin could be a therapeutic target in cancer treatment. Both peptides and monoclonal antibodies targeting N-cadherin have shown some efficacy in the preclinical setting. In an in vivo preclinical melanoma model, inhibition of N-cadherin function with a cyclic pentapeptide combined with chemotherapy led to enhanced tumor cell apoptosis and inhibition of tumor growth [42]. In another study, the activity of this cyclic pentapeptide N-cadherin inhibitor was determined in vivo to occur through altering AKT activity and altering vascular permeability [43]. In a similar model for prostate cancer, the same drug mimicked the results of N-cadherin ablation in prostate cancer cells, reducing immunosuppression and enhancing tumor-infiltrating leukocyte-related therapy by modulating the expression of immune checkpoint inhibitors [44].

In conclusion, we present novel insights into the reciprocal N-cadherin-N-cadherin interactions between melanoma cells and CAFs and how their interactions promote melanoma cell proliferation and tumor progression. However, it remains to be further explored to understand how the E- to N-cadherin switch and EMT in melanoma cells could be reversed by targeting N-cadherin in CAFs and/or melanoma cells. In addition, the mechanism that controls the recycling, internalization, degradation, and expression of N-cadherin in melanoma cells by YAP1 signaling in CAFs needs to be elucidated. Nevertheless, further exploration of YAP1 and N-cadherin as therapeutic targets to inhibit melanoma metastasis and improve targeted therapies and immunotherapies is warranted.

## Materials and methods

### Cell lines

The murine D4M melanoma cell line was purchased from Kerafast (Boston, MA) [45]. The human melanoma cell lines A375, SK-MEL-24, and 1205Lu were purchased from the American Type Culture Collection (ATCC, Manassas, VA) and maintained in Dulbecco’s modified Eagle medium (DMEM) in a humidified incubator at 37°C with 5% CO_2_. Two human CAF cell lines (224350P1/M50 and DT01027P1/M27) were isolated from surgically excised human melanoma tissues and obtained from Asterand Bioscience (Detroit, MI). All culture media were supplemented with 10% (v/v) fetal bovine serum (FBS) and 1% (v/v) 10,000 U/ml penicillin and 10,000 U/ml streptomycin. All cell culture reagents were purchased from ThermoFisher Scientific (Rochester, NY) unless otherwise stated. The isolation and maintenance of primary human fibroblasts were approved by the Institutional Review Board and the Institutional Biosafety Office of the University of Cincinnati. Experimental procedures involving biosafety issues were carried out under the University of Cincinnati Institutional Biosafety Committee protocol 16-08-17-01.

### Statistical analysis

All quantitative results were obtained from a minimum of three independent experiments. Data were analyzed using the GraphPad Prism 9 software package (GraphPad Software Inc., San Diego, CA) and expressed as the mean ± SD. The mean difference was determined by Student’s t-tests and considered statistically significant at *P* < 0.05.

### Supplementary information

Supplementary file 1

Supplementary file 2

## Data Availability

The data generated in this study are available within the article and its supplementary data files. RNA-Seq data generated in this study are publicly available in Gene Expression Omnibus (GEO) at GSE208058.

## Materials availability

All materials underlying this study are available from the corresponding author based on a material transfer agreement. RNA-Seq data were deposited in the GEO database (GSE208058).

## References

[1] Andl T, Zhou L, Yang K, Kadekaro AL, Zhang Y (2017). YAP and WWTR1: New targets for skin cancer treatment. Cancer Lett.

[2] Udan RS, Kango-Singh M, Nolo R, Tao C, Halder G (2003). Hippo promotes proliferation arrest and apoptosis in the Salvador/Warts pathway. Nat Cell Biol.

[3] Moroishi T, Hansen CG, Guan KL (2015). The emerging roles of YAP and TAZ in cancer. Nat Rev Cancer (Prog).

[4] Piersma B, de Rond S, Werker PM, Boo S, Hinz B, van Beuge MM (2015). YAP1 is a driver of myofibroblast differentiation in normal and diseased fibroblasts. Am J Pathol.

[5] Tschumperlin DJ, Liu F (2014). Pathologic matrix stiffness drives fibroblast activation through transcriptional regulators Yap and Taz. Am J Resp Crit Care.

[6] Liu T, Zhou L, Yang K, Iwasawa K, Kadekaro AL, Takebe T (2019). The beta-catenin/YAP signaling axis is a key regulator of melanoma-associated fibroblasts. Signal Transduct Target Ther.

[7] Lee MJ, Byun MR, Furutani-Seiki M, Hong JH, Jung HS (2014). YAP and TAZ regulate skin wound healing. J Invest Dermatol.

[8] Shiga K, Hara M, Nagasaki T, Sato T, Takahashi H, Takeyama H (2015). Cancer-associated fibroblasts: their characteristics and their roles in tumor growth. Cancers.

[9] Calvo F, Ege N, Grande-Garcia A, Hooper S, Jenkins RP, Chaudhry SI (2013). Mechanotransduction and YAP-dependent matrix remodelling is required for the generation and maintenance of cancer-associated fibroblasts. Nat Cell Biol.

[10] Maller O, DuFort CC, Weaver VM (2013). YAP forces fibroblasts to feel the tension. Nat Cell Biol (N. Views).

[11] Xin M, Kim Y, Sutherland LB, Murakami M, Qi X, McAnally J (2013). Hippo pathway effector Yap promotes cardiac regeneration. Proc Natl Acad Sci USA.

[12] Xin M, Kim Y, Sutherland LB, Qi X, McAnally J, Schwartz RJ (2011). Regulation of insulin-like growth factor signaling by Yap governs cardiomyocyte proliferation and embryonic heart size. Sci Signal.

[13] Wendling O, Bornert JM, Chambon P, Metzger D (2009). Efficient temporally-controlled targeted mutagenesis in smooth muscle cells of the adult mouse. Genesis.

[14] Choi SY, Sung R, Lee SJ, Lee TG, Kim N, Yoon SM (2013). Podoplanin, alpha-smooth muscle actin or S100A4 expressing cancer-associated fibroblasts are associated with different prognosis in colorectal cancers. J Korean Med Sci.

[15] Gao T, Zhou D, Yang C, Singh T, Penzo-Mendez A, Maddipati R (2013). Hippo signaling regulates differentiation and maintenance in the exocrine pancreas. Gastroenterology.

[16] Belteki G, Haigh J, Kabacs N, Haigh K, Sison K, Costantini F (2005). Conditional and inducible transgene expression in mice through the combinatorial use of Cre-mediated recombination and tetracycline induction. Nucleic Acids Res.

[17] Liu T, Zhou L, Xiao Y, Andl T, Zhang Y (2022). BRAF inhibitors reprogram cancer-associated fibroblasts to drive matrix remodeling and therapeutic escape in melanoma. Cancer Res.

[18] Seetharaman S, Etienne-Manneville S (2020). Cytoskeletal crosstalk in cell migration. Trends Cell Biol.

[19] Stylianou A, Gkretsi V, Louca M, Zacharia LC, Stylianopoulos T (2019). Collagen content and extracellular matrix cause cytoskeletal remodelling in pancreatic fibroblasts. J R Soc Interface.

[20] Mi H, Muruganujan A, Ebert D, Huang X, Thomas PD (2019). PANTHER version 14: more genomes, a new PANTHER GO-slim and improvements in enrichment analysis tools. Nucleic Acids Res.

[21] Kanehisa M, Goto S (2000). KEGG: kyoto encyclopedia of genes and genomes. Nucleic Acids Res.

[22] Kanehisa M, Furumichi M, Sato Y, Kawashima M, Ishiguro-Watanabe M (2023). KEGG for taxonomy-based analysis of pathways and genomes. Nucleic Acids Res.

[23] Radice GL (2013). N-cadherin-mediated adhesion and signaling from development to disease. Prog Mol Biol Transl Sci.

[24] Loh CY, Chai JY, Tang TF, Wong WF, Sethi G, Shanmugam MK (2019). The E-cadherin and N-cadherin switch in epithelial-to-mesenchymal transition: signaling, therapeutic implications, and challenges. Cells.

[25] Zhang J, Shemezis JR, McQuinn ER, Wang J, Sverdlov M, Chenn A (2013). AKT activation by N-cadherin regulates beta-catenin signaling and neuronal differentiation during cortical development. Neural Dev.

[26] Liu T, Zhou L, Li D, Andl T, Zhang Y. Cancer-associated fibroblasts build and secure the tumor microenvironment. Front Cell Dev Biol. 2019;7:60. 10.3389/fcell.2019.00060. 10.3389/fcell.2019.00060 PMC6492564 31106200

[27] Zhou L, Yang K, Dunaway S, Abdel-Malek Z, Andl T, Kadekaro AL (2018). Suppression of MAPK signaling in BRAF-activated PTEN-deficient melanoma by blocking beta-catenin signaling in cancer-associated fibroblasts. Pigment Cell Melanoma Res.

[28] Wheelock MJ, Shintani Y, Maeda M, Fukumoto Y, Johnson KR (2008). Cadherin switching. J Cell Sci.

[29] Mege RM, Ishiyama N (2017). Integration of cadherin adhesion and cytoskeleton at adherens junctions. Cold Spring Harb Perspect Biol.

[30] Zhang Y, Xie P, Wang X, Pan P, Wang Y, Zhang H (2018). YAP promotes migration and invasion of human glioma cells. J Mol Neurosci.

[31] Mui KL, Bae YH, Gao L, Liu SL, Xu T, Radice GL (2015). N-cadherin induction by ECM stiffness and FAK overrides the spreading requirement for proliferation of vascular smooth muscle cells. Cell Rep.

[32] Frixen UH, Behrens J, Sachs M, Eberle G, Voss B, Warda A (1991). E-cadherin-mediated cell-cell adhesion prevents invasiveness of human carcinoma cells. J Cell Biol.

[33] Islam S, Carey TE, Wolf GT, Wheelock MJ, Johnson KR (1996). Expression of N-cadherin by human squamous carcinoma cells induces a scattered fibroblastic phenotype with disrupted cell-cell adhesion. J Cell Biol.

[34] Hsu MY, Meier FE, Nesbit M, Hsu JY, Van Belle P, Elder DE, et al. E-cadherin expression in melanoma cells restores keratinocyte-mediated growth control and down-regulates expression of invasion-related adhesion receptors. Am J Pathol. 2000;156:1515–25. 10.1016/S0002-9440(10)65023-7 PMC1876923 10793063

[35] Li G, Satyamoorthy K, Herlyn M (2001). N-cadherin-mediated intercellular interactions promote survival and migration of melanoma cells. Cancer Res.

[36] Ciolczyk-Wierzbicka D, Laidler P (2018). The inhibition of invasion of human melanoma cells through N-cadherin knock-down. Med Oncol.

[37] Hao L, Ha JR, Kuzel P, Garcia E, Persad S (2012). Cadherin switch from E- to N-cadherin in melanoma progression is regulated by the PI3K/PTEN pathway through Twist and Snail. Br J Dermatol.

[38] Bruser L, Bogdan S (2017). Adherens junctions on the move-membrane trafficking of E-cadherin. Cold Spring Harb Perspect Biol.

[39] Lindsay AJ, McCaffrey MW (2017). Rab coupling protein mediated endosomal recycling of N-cadherin influences cell motility. Oncotarget.

[40] Moreno-Layseca P, Icha J, Hamidi H, Ivaska J (2019). Integrin trafficking in cells and tissues. Nat Cell Biol.

[41] Qian X, Anzovino A, Kim S, Suyama K, Yao J, Hulit J (2014). N-cadherin/FGFR promotes metastasis through epithelial-to-mesenchymal transition and stem/progenitor cell-like properties. Oncogene.

[42] Augustine CK, Yoshimoto Y, Gupta M, Zipfel PA, Selim MA, Febbo P (2008). Targeting N-cadherin enhances antitumor activity of cytotoxic therapies in melanoma treatment. Cancer Res.

[43] Turley RS, Tokuhisa Y, Toshimitsu H, Lidsky ME, Padussis JC, Fontanella A (2015). Targeting N-cadherin increases vascular permeability and differentially activates AKT in melanoma. Ann Surg.

[44] Sun Y, Jing J, Xu H, Xu L, Hu H, Tang C (2021). N-cadherin inhibitor creates a microenvironment that protect TILs from immune checkpoints and Treg cells. J Immunother Cancer.

[45] Jenkins MH, Steinberg SM, Alexander MP, Fisher JL, Ernstoff MS, Turk MJ (2014). Multiple murine BRaf(V600E) melanoma cell lines with sensitivity to PLX4032. Pigment Cell Melanoma Res.

