# Supplementary material for: YAP1 controls the N-cadherin-mediated tumor-stroma interaction in melanoma progression
Source: Oncogene. 2024 Feb 2;43(12):884–98. doi: 10.1038/s41388-024-02953-1 (PMC10942861; doi:10.1038/s41388-024-02953-1)
Supplement: Supplementary file 1 [file 41388_2024_2953_MOESM1_ESM.docx]

**Materials and methods**

**Mice**

The *α-SMA-CreER^T2^* mouse strain was generated and authorized by Dr. Pierre Chambon at The Institute of Genetics and Molecular and Cellular Biology (IGBMC) in France and kindly provided by Dr. Richard T. Premont at Duke University. The *Yap1^loxP/loxP^* mouse strain was generated and authorized by Dr. Eric Olson at UT Southwestern and kindly provided by Dr. Mei Xin at the Cincinnati Children’s Hospital Medical Center. C57BL/6J (B6), *Rosa-rtTA* (Jax 005670) and *tetO-Yap1* (Jax 031279) mouse strains were obtained from the Jackson Laboratory (Bar Harbor, ME, USA). Both mouse strains were backcrossed to the B6 background. *Yap1^loxP/loxP^* mice were crossed with *α-SMA-CreER^T2^* mice for several generations to generate *Yap1^loxP/loxP^; α-SMA-CreER^T2^* mice. *tetO-Yap1*, *α-SMA-CreER^T2^* and *Rosa-rtTA* mouse strains were crossed to generate triple *tetO-Yap1*;*α-SMA-CreER^T2^*; *Rosa-rtTA* mice. Mice were genotyped by polymerase chain reaction (PCR) analysis of genomic DNA extracted from tail biopsies. The *Yap1^loxP/loxP^* alleles were genotyped using the following primer pair: forward: 5′ ACATGTAGGTCTGATGCCAGAGGAGG 3′ and reverse: 5′ AGGCTGAGACAGGGGATCTCTGTGAG 3′. The *tetO-Yap1* alleles were genotyped using the following primer pair: forward: 5′ GCTGCCACCAAGCTAGATAAAG 3′ and reverse: 5′ CTGAACTTGTGGCCGTTTAC 3′. The *Rosa-rtTA* alleles were genotyped with the following primer pair: forward: 5′ AAGACCGCGAAGAGTTTGTC 3′ and reverse: 5′ AAAGTCGCTCTGAGTTGTTAT 3′. The presence of the Cre transgene (*α-SMA-CreER^T2^*) was identified by PCR using the following primer pair: forward: 5′ CGGTCTGGCAGTAAAAACTAT 3′ and reverse: 5′ CAGGGTGTTATAAGCAATCCC 3′. All mice were housed in the Laboratory Animal Services Facility of the University of Cincinnati under an artificial 12 h/12 h light-dark cycle and were allowed free access to food and water. The Institutional Animal Care and Use Committee of the University of Cincinnati (IACUC) approved all experimental procedures involving mice (22-08-19-01).

**Mouse melanoma induction**

To generate melanomas carrying YAP1-deficient CAFs in mice, as shown in Fig 1A, fibroblasts of genotype *α-SMA-CreER^T2^; Yap1^loxP/loxP^* and control *α-SMA-CreER^T2^; Yap1* were isolated from 2- or 3-day-old neonatal littermates without induction and validated by immunostaining for the expression of α-SMA, vimentin, S100A4, keratin 14, and TRP1. Subsequently, a mix of 2 x 10^5^ D4M melanoma cells and uninduced *α-SMA-CreER^T2^; Yap1^loxP/loxP^* or control *α-SMA-CreER^T2^; Yap1* fibroblasts at a ratio of 1:1 was injected intradermally into the flanks of 5- to 6-week-old mice possessing the same genotype as injected fibroblasts. Mice were monitored daily for tumor formation. To induce the activation of Cre recombinase and *Yap1* knockout in *α-SMA-CreER^T2^; Yap1^loxP/loxP^* fibroblasts, all mice underwent intraperitoneal injection of 10 mg/ml tamoxifen (Sigma‒Aldrich, St. Louis, MO) in corn oil at 1 mg/g body weight for seven consecutive days after tumors reached a volume of approximately 62.5 cubic millimeters (counted as day 1). Meanwhile, the size of the tumors was measured and recorded for three weeks. Mice were euthanized when the tumor size exceeded 20% of body size, and tumors were harvested for various analyses. The number of mice used in each study was calculated using power analysis. Littermates are randomly selected for each group. Specific randomization and blinding are not necessary for the reported study.

To generate melanomas carrying YAP1-overexpressing CAFs in mice, as shown in Fig 2A, fibroblasts of genotype *α-SMA-CreER^T2^*; *Rosa-rtTA; tetO-Yap1* and control *α-SMA-CreER^T2^*; *Rosa-rtTA* were isolated from 2- or 3-day-old neonatal littermates without induction and validated by immunostaining for the expression of α-SMA, vimentin, S100A4, keratin 14, and TRP1. Subsequently, a mix of 1X 10^5^ D4M melanoma cells and 1X 10^5^ uninduced *α-SMA-CreER^T2^*; *Rosa-rtTA; tetO-Yap1* or control *α-SMA-CreER^T2^*; *Rosa-rtTA* fibroblasts was injected intradermally into the flanks of mice carrying the same genotype as injected fibroblasts. Mice were monitored daily for tumor formation. To induce the activation of Cre recombinase and YAP1 overexpression in *α*-*SMA-CreER^T2^; Rosa-rtTA; tetO-Yap1* fibroblasts, all mice were fed a doxycycline (Dox) diet (Bio-Serv, F-4096) and underwent intraperitoneal injection of 10 mg/ml tamoxifen (Sigma‒Aldrich, St. Louis, MO) in corn oil at 1 mg/g body weight for seven consecutive days after tumors reached a volume of approximately 62.5 cubic millimeters (counted as day 1). Meanwhile, the size of the tumors was measured and recorded for three weeks. Mice were euthanized when tumor size exceeded 20% of body size, and tumors were harvested for various analyses.

**Melanoma cell isolation**

To isolate melanoma cells from mouse tumors, we labeled D4M melanoma cells with a red fluorescent protein (RFP) for easy identification and isolation. Fresh melanomas were excised from mice on day 21. After removing necrotic and connective tissues, the remaining tissues were minced using a scalpel blade into small pieces and incubated with 5 ml of 2.5 mg/ml collagenase IV at 37°C with stirring for one hour to generate a single-cell suspension. After digestion, the mixture was filtered through a 40 µm cell strainer to remove tissue debris and cell clumps. The filtered cell suspension was centrifuged at 1500 rpm for 10 minutes. Afterwards, the cell pellet was washed with DMEM medium with 10% FBS twice and then suspended in PBS solution with 2% FBS for fluorescence-activated cell sorting (FACS) to isolate RFP-tagged D4M cells.

**Melanoma xenografting experiment**

The human melanoma xenograft model was established using a combination of BRAF-mutant human melanoma cell line A375 and CAF cell line M50 with or without YAP1 expression as shown in Fig. S5. To induce melanoma in mice, a mixture of 1 X 10^6^ A375 cells and 1 X 10^6^ uninduced GFP/M50 or shYAP1-GFP/M50 in 50 µL of medium was injected intradermally into the flanks of 4- to 6-week-old NOD SCID mice (*NOD.Cg-Prkdc^scid^/J*, JAX 001303), respectively. Mice were monitored daily for tumor appearance. To induce YAP1 ablation, all mice were fed a Dox diet for four weeks after the tumors reached a volume size of approximately 62.5 cubic millimeters. The tumor sizes were measured and recorded every other day until the endpoint, when the tumor size exceeded 20% of the mouse body size. After mice were euthanized, tumors were harvested for various analyses as described. The number of mice used in the study was calculated using power analysis. Littermates are randomly selected for each group. Specific randomization and blinding are not necessary for study.

**Histology, immunofluorescence staining, and immunohistochemistry**

Tumor tissues were fixed in 10% formalin overnight at 4°C and embedded in paraffin. Five-micron-thick paraffin-embedded tumor tissue sections were prepared for hematoxylin and eosin (H&E) staining and immunostaining as described previously [1, 2]. For histological analysis, paraffin sections were stained using a standard H&E staining protocol. Slides were mounted using VectaMount permanent mounting medium (Vector lab, Burlingame, CA) and analyzed using a brightfield microscope.

For immunostaining, the following primary antibodies were used: anti-α-SMA (Invitrogen, A2547, 1:200), anti-fibronectin (Sigma‒Aldrich, F3648, 1:200), anti-Ki67 (BD Pharmingen, #550609, 1:50), anti-Cyclin D1 (Cell Signaling, #2978, 1:50), anti-N-cadherin (Abcam, ab18203, 1:100), anti-p-Akt (phospho S473, Abcam, ab66138, 1:100), anti-YAP1 (Proteintech, 13584-1-AP, 1:500), and anti-TE7 (Millipore, CBL271, 1:200). After incubation with primary antibodies, slides were washed with PBS three times, incubated with the corresponding biotin-conjugated secondary antibodies at room temperature for one hour, and then incubated with either fluorochrome-conjugated streptavidin for immunofluorescence or VECTASTAIN Elite ABC Reagents (Vector Laboratories, Burlingame, CA) for immunohistochemistry. Nuclei were counterstained with DAPI (blue) for immunofluorescence or hematoxylin (blue) for immunohistochemistry. Images were taken using a Nikon Eclipse 80i fluorescence microscope. The number of positively stained cells in each high-power field (40X) was counted using the particle analysis function of ImageJ software (NIH). The number of positive cells per square millimeter was calculated by multiplying the number of cells counted in each field by 4.5. The percentage of the surface area occupied by α-SMA+ cells in each high-power field (40X) was measured using ImageJ and calculated by dividing by the area of the entire field.

**Collagen staining**

The collagen content in melanoma tissues was evaluated using the Picrosirius Red staining kit (American MasterTech, Lodi, CA) according to the manufacturer’s instructions. After staining, the slides were washed with 1% acetic acid for one minute, dehydrated in 100% ethanol, cleared using xylene, and mounted using VectaMount permanent mounting medium.

**Quantification of collagen content**

To quantify collagen content in melanoma tissues, a Sirius Red/Fast Green Collagen Staining Kit (Chondrex, Redmond, WA) was used according to the manufacturer’s instructions. Briefly, after the slide was incubated with the dye solution at room temperature for 30 minutes, the dye solution was removed, and the slide was rinsed with distilled water until the water became colorless. One milliliter of dye extraction buffer was added to each slide to elute the dye from dyed tissues. A 200 μl dye extraction solution from each slide was collected for absorbance measurement at 540 nm and 605 nm using a microplate reader. The collagen content in each sample was normalized to the total protein content.

**Generation of inducible YAP1-deficient CAFs**

To ablate YAP1 expression in CAFs, M27, and M50 were transduced with doxycycline-dependent inducible lentivirus expressing shRNAs that specifically target YAP1 expression (V3SH7669-225152043, V3SH7669-225222498, V3SH7669-226435710 from Horizon Discovery, Lafayette, CO). A nontargeting shRNA lentivirus was used to generate control M27 and M50. The inducible lentiviral shRNA vector, which uses the Tet-On inducible system, only allows the expression of YAP1-targeting shRNA or nontargeting shRNA when cells are treated with doxycycline. The expression of GFP is driven by the same tetO promoter so that the transduction and shRNA expression upon doxycycline treatment can be visually tracked by green fluorescence. Briefly, CAFs were seeded in 6-well tissue culture plates. When the cells reached 50% confluence, lentiviral particles were added and incubated with the cells for 16 hours. The medium containing viral particles was then replaced with fresh DMEM supplemented with 0.5% FBS. To select transduced fibroblasts, a medium containing 10 µg/ml puromycin was added and maintained for three days. To assess whether puromycin selection was complete, 500 ng/ml doxycycline (Fisher Scientific, Pittsburgh, PA) was added for 72 hours to induce the expression of shRNAs and GFP. The efficiency of inhibiting YAP1 expression by shRNA was determined by Western blotting and qPCR.

**N-cadherin siRNA silencing**

Silencer siRNAs targeting N-cadherin (siRNA ID: S2771 and S2773) and scramble Silencer® siRNA control were purchased from Thermo Fisher Scientific (Thermo Fisher Scientific, Rochester, NY). Melanoma cells and CAFs were seeded in 6-cm dishes at an initial cell density of 1 x 10^5^ cells, cultured for 24 hours until 60-70% confluence, and then transfected with siRNA using Lipofectamine RNAiMAX (Thermo Fisher Scientific, Rochester, NY). According to the manufacturer’s protocol, N-cadherin siRNA (10 µM) was diluted in 250 µl of Opti-MEM I reduced serum medium (Thermo Fisher Scientific, Rochester, NY) and mixed with 15 µl of Lipofectamine RNAiMAX in 250 µl of Opti-MEM I Reduced Serum Medium. After incubation at room temperature for 10 minutes, the mixture was added to the cells and incubated for three days. Afterward, the medium containing siRNA and RNAiMAX was replaced with a regular DMEM culture medium.

**Generation of inducible YAP1-deficient N-cadherin-overexpressing CAFs and N-cadherin-overexpressing melanoma cells**

To overexpress N-cadherin in YAP1-deficient CAFs and melanoma cells, we constructed a doxycycline-dependent inducible lentiviral construct expressing N-cadherin, pLV-Bsd-TRE3G-hCDH2, at VectorBuilder. Inc (Chicago, IL). shYAP1-GFP/M50 and A375 were co-transduced with lentivirus particles expressing N-cadherin and the Tet transactivator (TET3G). TET3G is expressed using a lentiviral construct, pLV-CMV>Tet3G/Hygro, provided by VectorBuilder (Chicago, IL). Briefly, CAFs and melanoma cells were seeded in 6-well tissue culture plates. When the cells reached 50% confluence, lentiviral particles were added and incubated with the cells for 16 hours. The medium containing viral particles was then replaced with fresh DMEM supplemented with 0.5% FBS. To select transduced cells, a medium containing 200 µg/ml hygromycin and 10 µg/ml blasticidin was added and maintained for three days. To assess whether the selection was complete, 500 ng/ml doxycycline (Fisher Scientific, Pittsburgh, PA) was added for 72 hours to induce the overexpression of N-cadherin. The efficiency of overexpressing N-cadherin was determined by Western blotting. N-cadherin-overexpressing A375 cells were named as A375-Ncad. YAP1-deficient shYAP1-GFP/M50 cells were named as shYAP1-GFP/M50-Ncad.

**Human melanoma cell labeling**

The human melanoma cell lines A375 and SK-MEL-24 were transduced with a nontargeting control hEF1α-TurboRFP lentivirus expressing RFP (GE Dharmacon, cat# VSC6573). Lentivirus transduction was carried out as described above. RFP expression and transduction efficiency were confirmed by flow cytometry.

**Chamber slide immunofluorescence staining**

1.5 x 10^4^ cells were counted and seeded in one well of an 8-well Nunc™ Lab-Tek™ II chamber slide (Thermo Fisher Scientific, Rochester, NY). The cells were fixed in 4% paraformaldehyde for 10 minutes at room temperature and then permeabilized using 0.05% Triton-100 for 10 minutes on ice for immunofluorescence staining. After permeabilization, the cells were washed three times with PBS and blocked in 10% normal goat serum for 1 hour at room temperature. Primary antibodies recognizing F-actin (Thermo Fisher, R415, 1:60), paxillin (BD Biosciences, 610051, 1:200), fibronectin (Sigma, F3648, 1:200), α-SMA (Thermo Fisher, 14–9760–82, 1:200), S100A4 (Thermo Fisher, 16105-1-AP, 1:200), N-cadherin (Abcam, ab18203, 1:100), MYH10 (Cell Signaling Technology, 3404, 1:100), and MLC2 (Cell Signaling Technology, 3672, 1:100) were then added to each specific chamber and incubated overnight at 4°C. The next day, after washing with PBS three times, an Alexa Fluor 488- or 594-conjugated secondary antibody (Thermo Fisher Scientific, Rochester, NY) was added to the corresponding well for an one-hour incubation at room temperature. The slides were mounted with VECTASHIELD Antifade Mounting Medium (Vector Laboratories, Burlingame, CA) and coverslipped. Images were acquired using a Nikon Eclipse 80i fluorescence microscope. For EdU staining, a Click-iT™ Plus EdU Cell Proliferation Kit was used for imaging according to the manufacturer’s instructions. The number of EdU+ cells and total cell number in each high-power field (40X) were counted using the particle analysis function of ImageJ software. The TUNEL assay was performed using an *In Situ* Cell Death Detection Kit following the standard protocol provided by the manufacturer (Sigma, St. Louis, MO).

**Cell viability and proliferation assays**

For cell number counting, 1 x 10^5^ cells were seeded in one 6-cm dish and cultured for seven days. The cells were collected on days 1, 3, 5, and 7 for cell number counting using a hemocytometer. At least three repeats were performed for each indicated cell line per assay, and cell number counting was performed a minimum of three times. The MTT assay was performed as we previously published [2].

**Generation of melanoma cell-conditioned culture medium**

To prepare melanoma cell-conditioned culture medium, A375 or SK-MEL-24 melanoma cells were seeded in 10 cm dishes. After the cells reached confluency, the medium was replaced with a serum-free medium for another 24-hour culture. Afterward, the medium was collected and centrifuged at 1000 × g for 10 minutes to remove cell debris for future use.

**Transwell migration assay**

To perform the transwell migration assay, 3.5 x 10^5^ CAFs were seeded on the insert with a permeable membrane (Greiner Bio-One, Kremsmünster, Austria) in a 6-well plate. To assess the response and migratory ability of CAFs to different chemoattractants with and without YAP1 expression, melanoma cell-conditioned medium was added to the bottom well, or melanoma cells were seeded in the well, ensuring that CAFs on the membrane were in contact with the medium. After 48 hours, CAFs that migrated through the membrane were stained and imaged. Because CAFs were transduced with GFP, migrated CAFs on the opposite side of the insert were first captured using a Cytation 1 cell imaging multimode reader (BioTek, Winooski, VT) and then fixed with paraformaldehyde for crystal violet staining. The migration area was defined as the area occupied by green fluorescent CAFs and was quantified using ImageJ.

**Collagen gel contraction assay**

A collagen gel contraction assay was performed as reported previously [1-3]. After 72 hours of doxycycline induction, 1 x 10^5^ CAF cells were resuspended in 500 µl of 1 mg/ml collagen solution and transferred into one well of a 24-well tissue culture plate. After a 30-minute incubation in a humidified incubator at 37°C, one ml of fresh medium was added on top of the gel for a 72-hour incubation. Afterward, gels were detached from the wall of each well and allowed to contract as indicated. Pictures of the gels were taken using a Nikon digital camera every 24 hours, and ZEN 2.3 software was used to measure the diameters. The gel contraction percentage was calculated by dividing the difference in gel diameters between 0 and 72 hours by the diameter at 0 hour.

**Confocal reflection microscopy (CRM)**

CRM was performed as reported previously [1-3]. After a 72-hour incubation, collagen fiber distribution was assessed using a Zeiss LSM 710 confocal microscope at 40X magnification. Images were acquired at a depth of at least 100 mm inside the gel to avoid edge effects. Pictures of at least ten areas of each gel were randomly captured for 3D reconstruction of the matrix using ImageJ. Fiber connectivity and spacing were calculated using ImageJ with the BoneJ plugin (<http://bonej.org>).

**3D invasion assay**

Collagen gels were prepared as described in the gel contraction assay. 100 μl of gel mixture was placed on the transwell insert in a 24-well plate and allowed to solidify for 30 minutes. Afterward, 5 x 10^4^ CAFs were resuspended in 300 μl of gel mixture and added on top of the solidified gel, and one ml of medium was added for incubation overnight. After 24 hours, the medium was removed, and 5 x 10^4^ A375 melanoma cells suspended in 100 μl medium were added on top of collagen gel embedded with CAFs. To allow melanoma cells to invade the underlying gel, gel assemblies carrying CAFs and A375 melanoma cells in 24-well plates were placed in an incubator for up to 15 days. On days 10 and 15, collagen gels were removed and fixed with 4% paraformaldehyde overnight at 4°C. Fixed gels were collected for paraffin section preparation and histological analysis to determine the distance of melanoma cell invasion in the matrices. The collagen content was determined as described above. The distance of melanoma cell invasion was measured using ImageJ.

**Quantitative real-time PCR assay**

RNA was extracted from cultured cells and melanoma tissues using a PurelinkTM RNA Mini kit (Thermo Fisher, Waltham, MA) according to the manufacturer’s protocol. For 3D coculture spheroids, RNA was extracted using an RNeasy Plus Micro Kit (Qiagen, Hilden, Germany). Briefly, frozen tumor tissues were added to an RNase-free mortar containing liquid nitrogen and ground thoroughly into powder using an RNase-free pestle before being transferred to an RNase-free microcentrifuge tube. Based on the weight of the powder, enough lysis buffer was added for the subsequent homogenization and RNA extraction steps. For cultured cells and spheroids, samples were collected and washed once using ice-cold PBS for RNA extraction. RNA concentration was determined using a NanoDrop spectrophotometer. cDNAs were generated by reverse transcription using a SuperScript IV first-strand synthesis system (Thermo Fisher, Waltham, MA). qPCRs were performed using SYPR green master mix power on a StepOnePlus real-time PCR system (Applied Biosystems, Waltham, MA). qPCR primers for fibronectin, tenascin C, talin, and N-cadherin were purchased from realtimeprimers.com (Philadelphia, PA). The relative expression level of each gene was normalized to the level of GAPDH. The data shown were generated from at least three independent repeats.

**3D spheroid cell coculture**

Co-cultured cell spheroids were formed by mixing 3000 RFP-tagged melanoma cells with 3000 CAFs in a total volume of 100 μl in 96-well plates (Thermo Fisher Scientific, Rochester, NY) with a low cell-adhesion surface. Fluorescent images of spheroids were taken every 24 hours for up to 72 hours using a Cytation 1 cell imaging multimode reader [3]. The RFP area and intensity were recorded each time. To count the RFP+ melanoma cell number in the spheroids, 12 spheroids from each group were collected and digested using 2 mg/ml collagenase IV (Thermo Fisher Scientific, MA) for 30 minutes at 37°C with stirring to generate a single cell suspension. A Countess II Automated Cell Counter (Thermo Fisher Scientific, Rochester, NY) was used to quantify the RFP+ melanoma cell number. The average melanoma cell number in each spheroid was calculated by dividing the total RFP+ melanoma cell number by 12. For immunofluorescence staining, spheroids were washed once with cold PBS, fixed in 4% paraformaldehyde, and dehydrated using ethanol. After dehydration, spheroids were embedded in 1% agarose gel for paraffin section preparation and immunostaining.

**RNA-Seq**

Total RNA was extracted from GFP/M50 and shYAP1-GFP/M50 cells after a 3-day doxycycline induction using a PureLink^TM^ RNA Mini Kit. RNA-Seq was performed as single-end sequencing at the University of Cincinnati Genomics, Epigenomics and Sequencing core using the Illumina NextSeq 550 system. Generated Fastq files were validated, processed, and analyzed using the UCSC human hg19 reference genome on the A.I.R. platform (Sequentia Biotech, Barcelona, Spain). Differentially expressed genes were identified using the DESeq2 package [4]. Genes with an adjusted p-value < 0.05 and a fold change > or < 2 were considered significant. Heatmaps of differentially expressed genes were generated using A.I.R. A volcano plot to visualize significant gene expression was generated using the OmicStudio tools [5]. Gene Ontology (GO) functional enrichment analysis was performed using A.I.R. Kyoto Encyclopedia of Genes and Genomes (KEGG) pathway enrichment analysis was performed using DAVID Bioinformatics Resources 6.8 [6]. The most affected sets of genes in YAP1-GFP/M50 upon YAP1 ablation were determined using the GSEA software package 4.3.1 [7].

**Western Blotting**

Standard Western blotting was performed as previously reported [1-3]. The following antibodies were used: anti-YAP1 (Proteintech, 13584-1-AP, 1:2500), anti-N-cadherin (Abcam, ab18203, 1:500), anti-Akt (Cell Signaling, #2920S, 1:1000), anti-p-Akt (S473) (Cell Signaling, #4060S, 1:1000), anti-MYH9 (Cell Signaling, 3403, 1:1000), anti-MYH10 (Cell Signaling, 8824, 1:1000), anti-MLC2 (Cell Signaling, 3672, 1:1000), anti-β-actin (Thermo Fisher, MA5-15739, 1:3000), anti-β-actin (Proteintech, MA5-15739, 1:3000), anti-β-tubulin (Thermo Fisher, MA5-16308, 1:3000), and anti-GAPDH (Thermo Fisher, 437000, 1:1000). For chemiluminescent Western blotting using X-ray film, the blots were incubated with either HRP-conjugated goat anti-mouse IgG (H+L) antibody (Cell Signaling, #7076, 1:2000) or HRP-conjugated goat anti-rabbit IgG (H+L) antibody (Cell Signaling, #7074, 1:1000) at room temperature for one hour. Afterward, the membranes were washed and incubated with ECL Western blotting detection substrate (GE Healthcare, Chicago, IL). for five minutes. The blots were then exposed to X-ray film. For fluorescent Western blotting using the Odyssey imaging systems, membranes were incubated with either IRDye 680RD goat anti-rabbit IgG secondary antibody (LI-COR, 926-68071, 1:2000), IRDye 800CW donkey anti-rabbit IgG secondary antibody (LI-COR, 926-32213, 1:2000), IRDye 680RD donkey anti-mouse IgG secondary antibody (LI-COR, 926-68072, 1:2000), or IRDye 800CW goat anti-mouse IgG secondary antibody (LI-COR, 926-32210, 1:2000) at room temperature for one hour. The membranes were then washed again using TBST and processed for scanning, visualization, and quantification using the Odyssey CLx imaging system (LI-COR, model #9140).

**Reference**

1 Zhou L, Yang K, Dunaway S, Abdel-Malek Z, Andl T, Kadekaro AL, Zhang Y. Suppression of MAPK signaling in BRAF-activated PTEN-deficient melanoma by blocking beta-catenin signaling in cancer-associated fibroblasts. *Pigment Cell Melanoma Res* 2018; 31: 297-307.

2 Liu T, Zhou L, Xiao Y, Andl T, Zhang Y. BRAF Inhibitors Reprogram Cancer-Associated Fibroblasts to Drive Matrix Remodeling and Therapeutic Escape in Melanoma. *Cancer Res* 2022; 82: 419-432.

3 Liu T, Zhou L, Yang K, Iwasawa K, Kadekaro AL, Takebe T *et al*. The beta-catenin/YAP signaling axis is a key regulator of melanoma-associated fibroblasts. *Signal Transduct Target Ther* 2019; 4: 63.

4 Love MI, Huber W, Anders S. Moderated estimation of fold change and dispersion for RNA-seq data with DESeq2. *Genome biology* 2014; 15: 550.

5 Lyu F, Han F, Ge C, Mao W, Chen L, Hu H *et al*. OmicStudio: A composable bioinformatics cloud platform with real‐time feedback that can generate high‐quality graphs for publication. *iMeta* 2023; 2.

6 Huang da W, Sherman BT, Lempicki RA. Systematic and integrative analysis of large gene lists using DAVID bioinformatics resources. *Nat Protoc* 2009; 4: 44-57.

7 Mootha VK, Lindgren CM, Eriksson KF, Subramanian A, Sihag S, Lehar J *et al*. PGC-1alpha-responsive genes involved in oxidative phosphorylation are coordinately downregulated in human diabetes. *Nat Genet* 2003; 34: 267-273.
