# Supplementary material for: YAP1 controls the N-cadherin-mediated tumor-stroma interaction in melanoma progression
Source: Oncogene. 2024 Feb 2;43(12):884–98. doi: 10.1038/s41388-024-02953-1 (PMC10942861; doi:10.1038/s41388-024-02953-1)
Supplement: Supplementary file 2 [file 41388_2024_2953_MOESM2_ESM.docx]

**
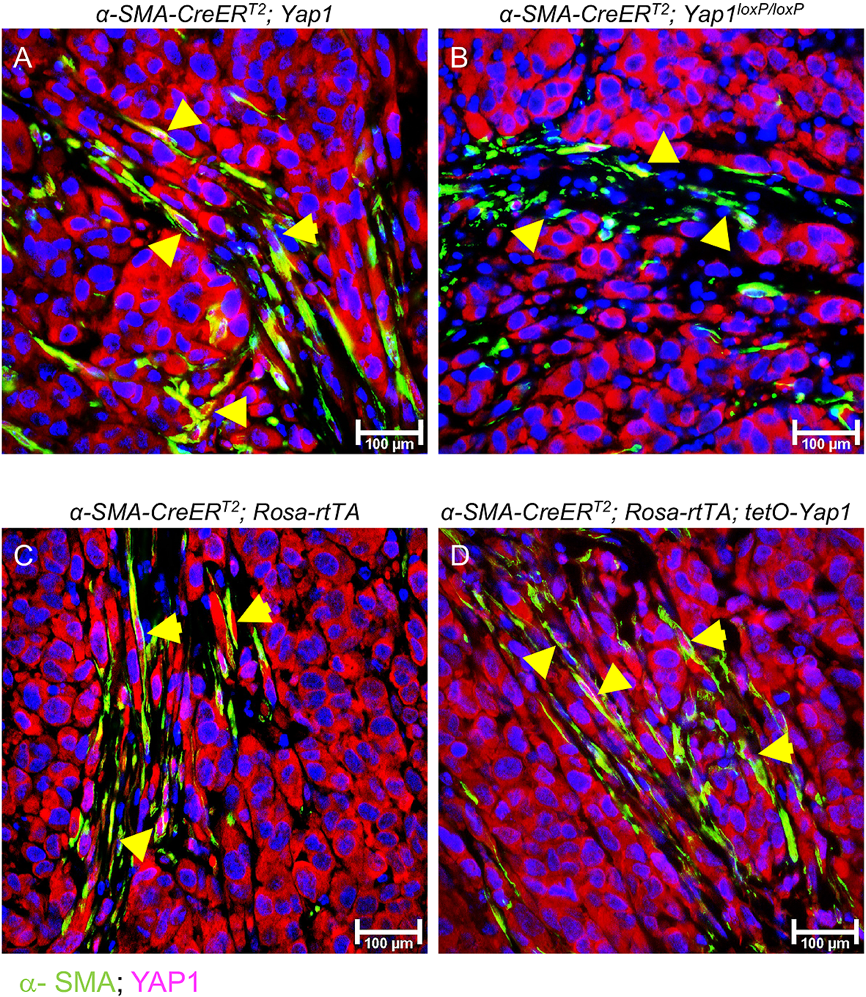
**

**Figure S1. YAP1 expression in mouse melanomas containing YAP1-deficient CAFs or YAP1-overexpressing CAFs.** Representative images show the expression of YAP1 in CAFs in the indicated mouse melanoma tissues visualized by co-immunostaining using an anti-α-SMA antibody (green) and an anti-YAP1 antibody (red). Yellow arrows indicate α-SMA+ CAFs. A-B. Mouse melanoma tissues containing YAP1-deficient fibroblasts (B) and control wildtype fibroblasts (A). C-D. Mouse melanoma tissues containing YAP1-overexpressing fibroblasts (D) and control wildtype fibroblasts (C).

**
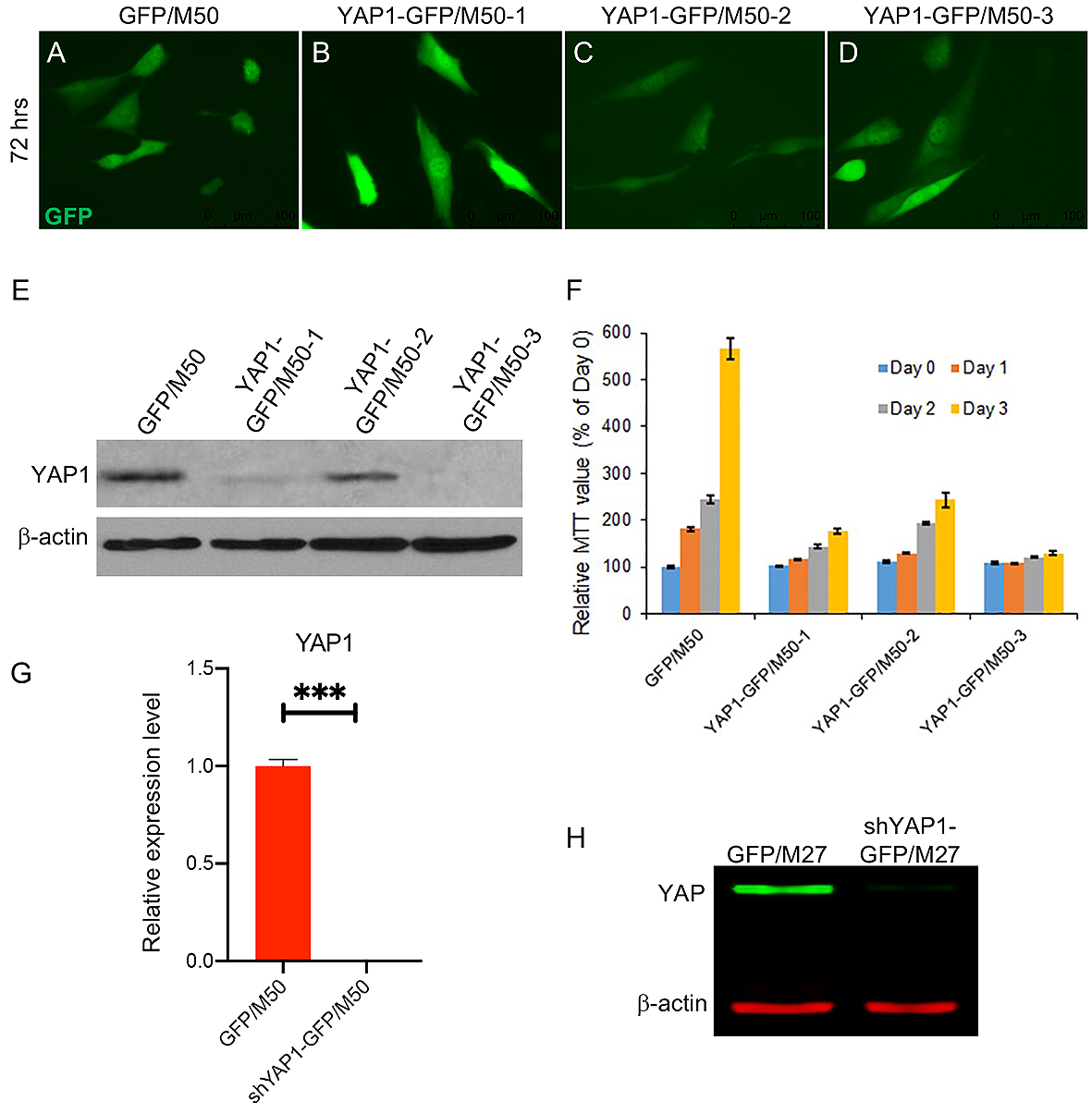
**

**Figure S2. YAP1 expression is silenced in human CAFs using shRNA.** Three inducible shRNAs were tested to silence YAP1 expression in CAFs. A-D. Images of GFP expression in M50 after the cells were transduced with nontargeting shRNA and three shRNAs and named GFP/M50, YAP1-GFP/M50-1, YAP1-GFP/M50-2, and YAP1-GFP/M50-3. E. Western blot shows the efficiency of YAP1 silencing in M50 after a 72-hour induction using three shRNAs. F. The viabilities of GFP/M50, YAP1-GFP/M50-1, YAP1-GFP/M50-2 and YAP1-GFP/M50-3 cells were compared using the MTT assay at day 1, 2, and 3. n=3. G: Quantitative analysis of YAP1 expression in GFP/M50 and YAP1-GFP/M50 cells by qPCR. H. Western blots show the loss of YAP expression in YAP1-GFP/M27 compared to GFP/M27.


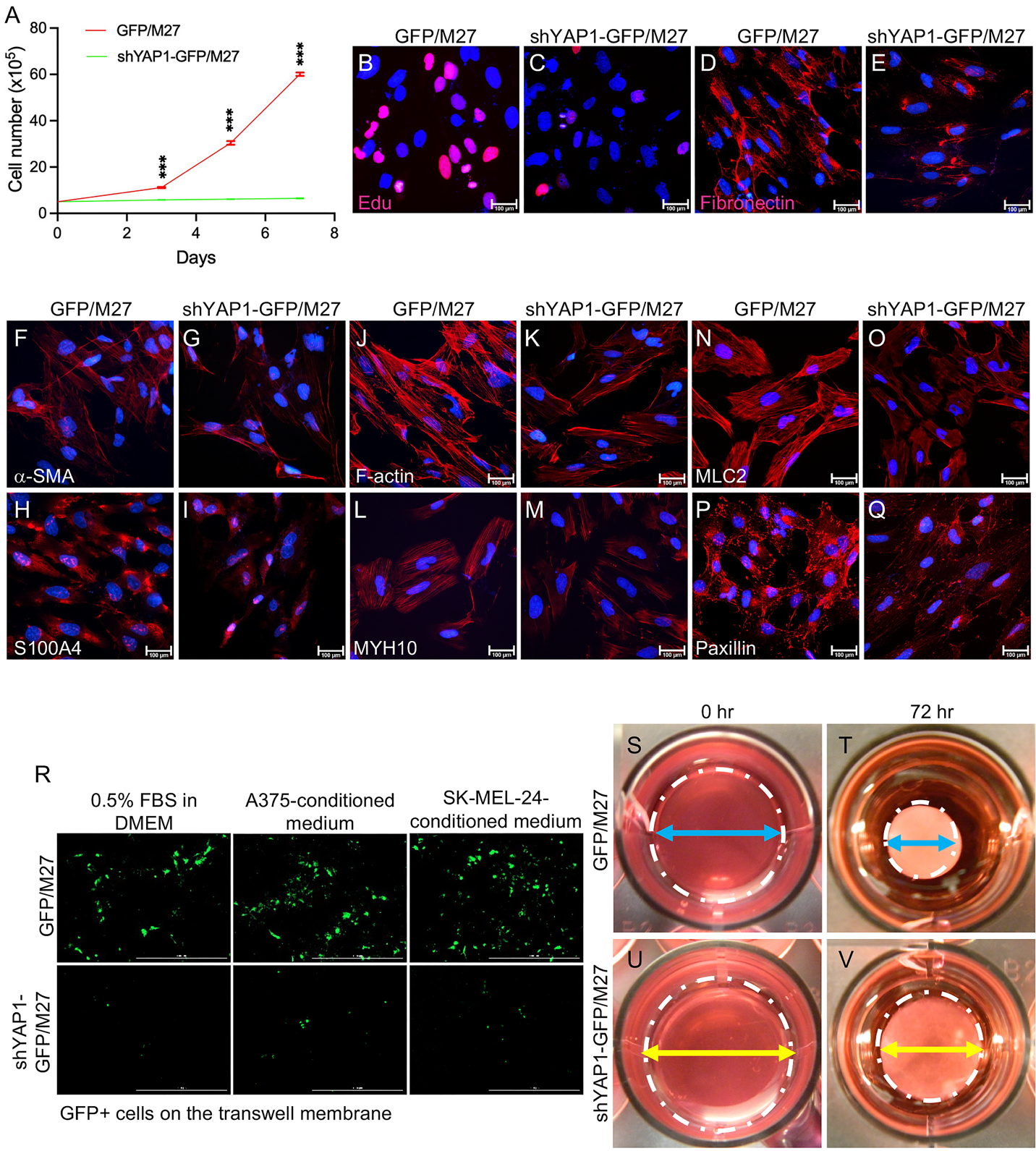


**Figure S3. YAP1 is essential for the functional properties of CAFs**. YAP1 in M27 was ablated using YAP1-GFP/Fb-3 shRNA (V3SH7669-226435710) and named as shYAP1-GFP/M27. The cells were treated with 500 ng/ml doxycycline for 72 hours for indicated experiments unless otherwise stated. A. Comparison of the numbers of GFP/M27 and shYAP1-GFP/M27 in culture for seven days. n=3. The data are represented as the mean ± SD. *, P≤0.05; **, P≤0.01; ***, P≤0.001; ns, not significant. B-C. Images show EdU staining of GFP/M27 and shYAP1-GFP/M27 with blue DAPI nuclear counterstaining. D-E. Images show fibronectin staining of GFP/M27 and shYAP1-GFP/M27 with blue DAPI nuclear counterstaining. F-Q. Images show α-SMA, S100A4, F-actin, MYH10, MLC2, and paxillin staining of GFP/M27 and shYAP1-GFP/M27 with blue DAPI nuclear counterstaining. R. Images show the migratory response of GFP/M27 and YAP1-GFP/M27 cultured in DMEM with 0.5% FBS, A375-conditioned medium, and SK-MEL-24-conditioned medium at 48 hours using the transwell migration assay. S-V. Representative images of contracted collagen gels embedded with GFP/M27 and shYAP1-GFP/M27 at 0 and 72 hours. The gel in each well is circled with a white dashed line. The blue and yellow arrow lines indicate the diameter of the contracted gels embedded with GFP/M27 and shYAP1-GFP/M27, respectively. For all staining pictures, the scale bar represents 100 µm. Each immunostaining was repeated for a minimum of three times.

**
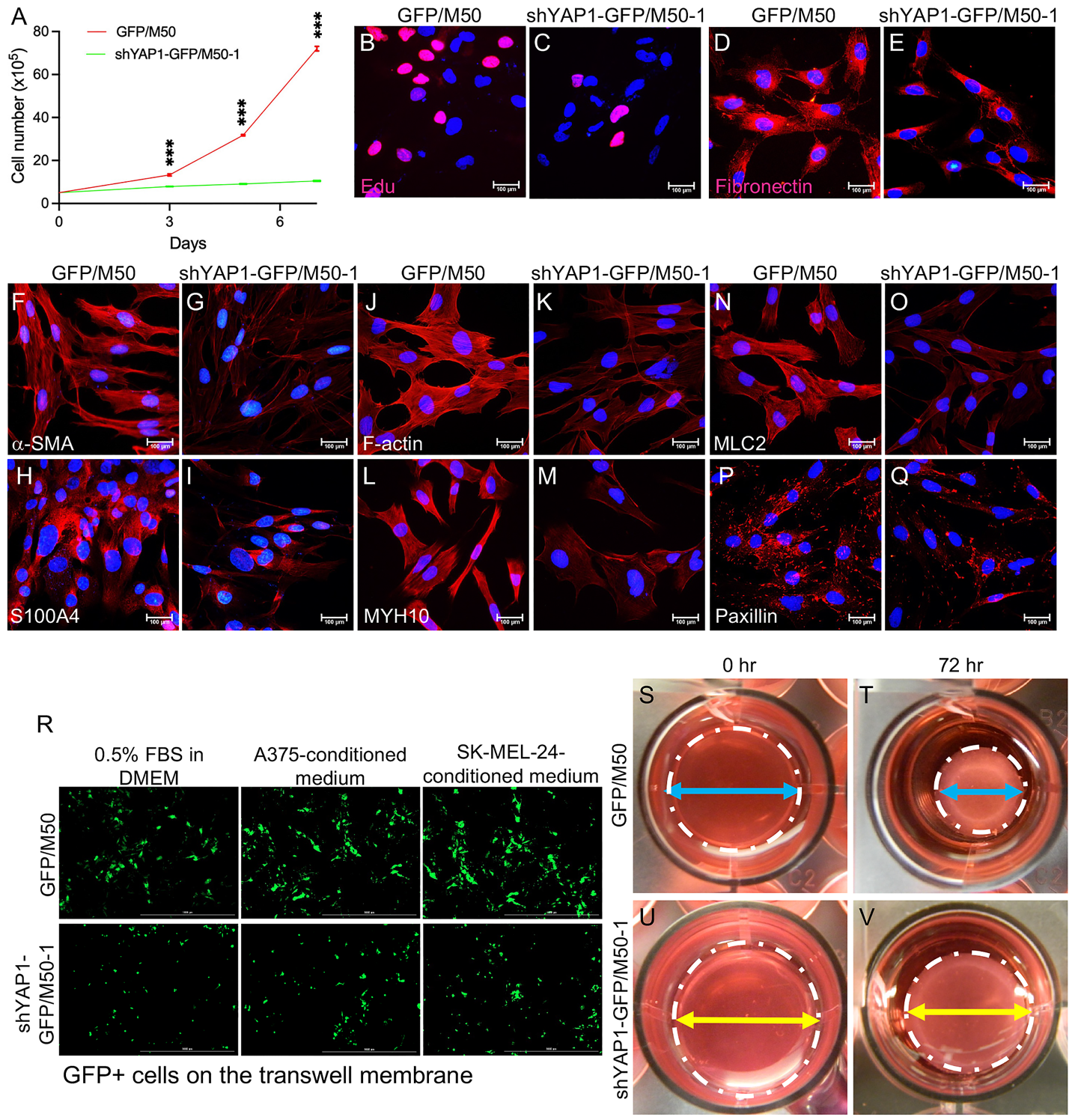
Figure S4. YAP1 is essential for the functional properties of CAFs**. YAP1 in M50 was ablated using YAP1-GFP/Fb-1 shRNA (V3SH7669-225222498) and named as shYAP1-GFP/M50-1. The cells were treated with 500 ng/ml doxycycline for 72 hours for indicated experiments unless otherwise stated. A. Comparison of the numbers of GFP/M50 and shYAP1-GFP/M50-1 in culture for seven days. n=3. The data are represented as the mean ± SD. *, P≤0.05; **, P≤0.01; ***, P≤0.001; ns, not significant. B-C. Images show EdU staining of GFP/M50 and YAP1-GFP/M50-1 with blue DAPI nuclear counterstaining. D-E. Images show fibronectin staining of GFP/M50 and shYAP1-GFP/M50-1 with blue DAPI nuclear counterstaining. F-Q. Images show α-SMA, S100A4, F-actin, MYH10, MLC2, and paxillin staining of GFP/M50 and shYAP1-GFP/M50-1 with blue DAPI nuclear counterstaining. R. Images show the migratory response of GFP/M50 and shYAP1-GFP/M50-1 cultured in DMEM with 0.5% FBS, A375-conditioned medium, and SK-MEL-24-conditioned medium at 48 hours using the transwell migration assay. S-V. Representative images of contracted collagen gels embedded with GFP/M50 and shYAP1-GFP/M50-1 at 0 and 72 hours. The gel in each well is circled with a white dashed line. The blue and yellow arrow lines indicate the diameter of the contracted gels embedded with GFP/M50 and shYAP1-GFP/M50-1, respectively. For all staining pictures, the scale bar represents 100 µm. Each immunostaining was repeated for a minimum of three times.

**
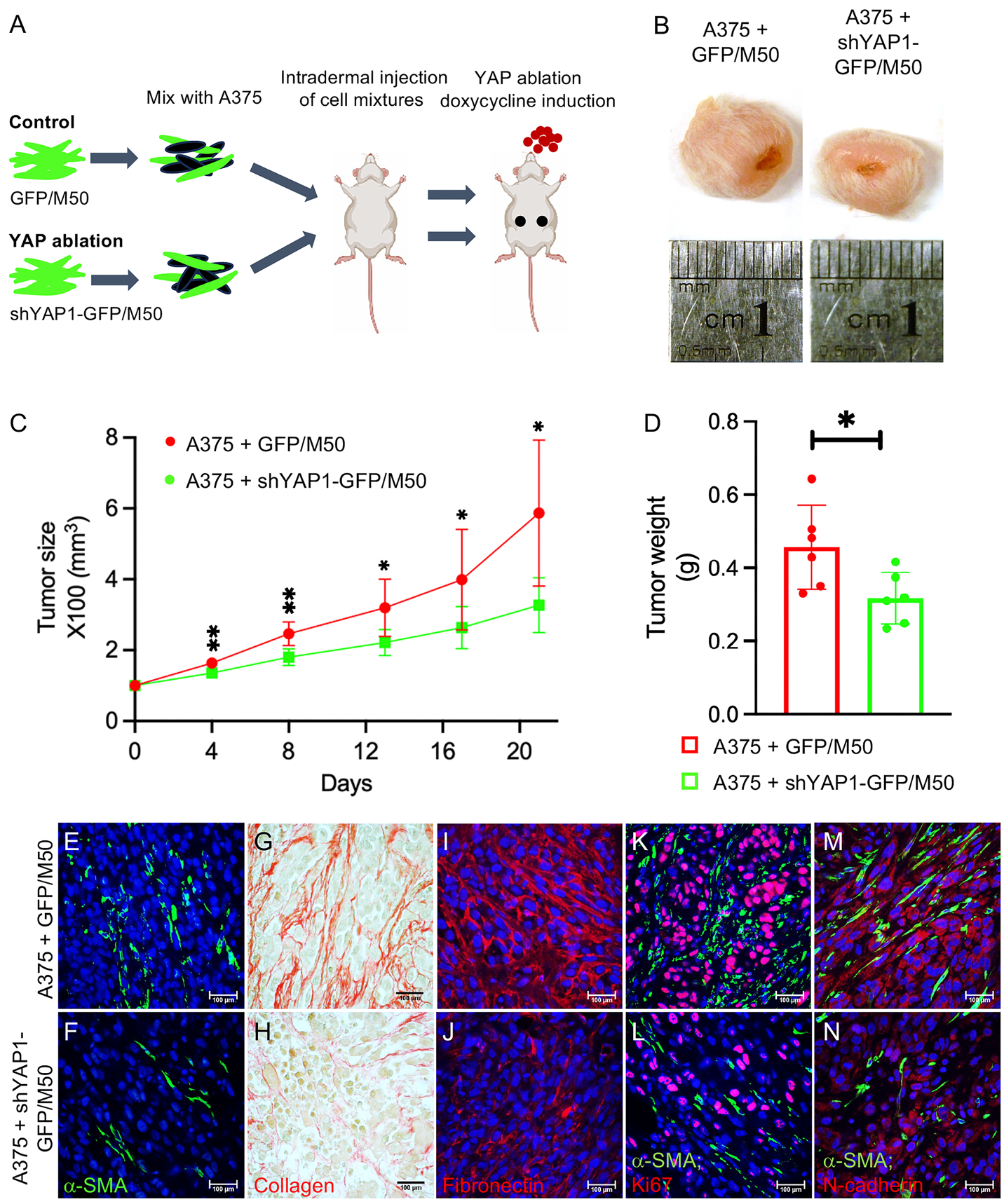
Figure S5. Human melanoma progression is suppressed by YAP1 ablation in CAFs**. A. Illustration of human melanoma xenograft model for studying the *in vivo* effects of YAP1 ablation in CAFs on melanoma progression. B. Representative pictures of melanomas formed by A375 and GFP/M50 or shYAP1-GFP/M50 on day 21. C. Tumor sizes were measured and compared between A375 + GFP/M50 melanomas and A375 + shYAP1-GFP/M50 melanomas on each indicated day after the induction of YAP1 ablation. n=6 for each time point. D. Tumor weight comparison between A375 + GFP/M50 melanomas and A375 + shYAP1-GFP/M50 melanomas on day 21. n=6. The box indicates the average tumor weight, and error bars indicate the mean ± SD. E-F. Fluorescence images show α-SMA staining of melanomas as indicated. The nuclei were counterstained using DAPI (blue). G-H. Images show collagen staining of melanoma tissue sections as indicated. I-J. Fluorescence images show ECM protein fibronectin expression in melanomas as indicated. K-L. Images show α-SMA and Ki67 co-staining of melanoma tissue sections as indicated. M-N. Representative images of α-SMA and N-cadherin co-staining of melanoma tissue sections as indicated. For all staining pictures, the scale bar represents 100 µm. In all statistical graphs, data are represented as the mean ± SD. *, P≤0.05; **, P≤0.01; ***, P≤0.001; ns, not significant. Each immunostaining was repeated for a minimum of three times.

**
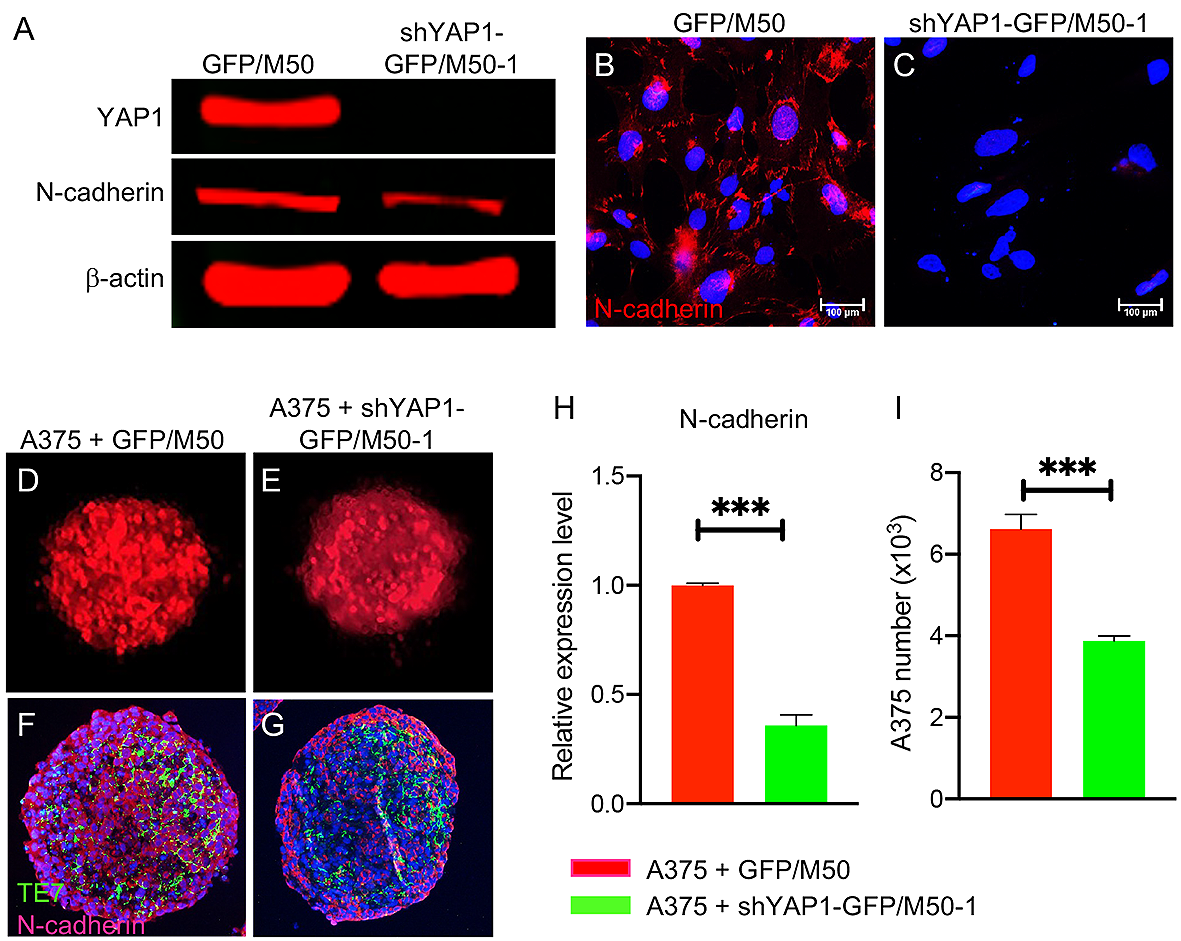
Figure S6. YAP1 deficiency-induced N-cadherin loss in CAFs leads to the downregulation of N-cadherin in melanoma cells**. A. Western blot shows that silencing YAP1 expression in M50 cells using YAP1-GFP/Fb-1 shRNA (V3SH7669-225222498) led to the loss of N-cadherin. B-C. Fluorescence images show the loss of N-cadherin in shYAP1-GFP/M50-1 by immunofluorescent staining. D-E. Fluorescence images show RFP-tagged A375 cells in spheroids co-cultured with GFP/M50 and shYAP1-GFP/M50-1. F-G. Fluorescence images show TE7 and N-cadherin co-staining of co-cultured spheroids as indicated using an anti-TE7 antibody (green) and an anti-N-cadherin antibody (red). H. Quantitative analysis of N-cadherin expression in co-cultured spheroids as indicated by qPCR. n=3. I. Graph shows the numbers of A375 cells in spheroids cocultured with GFP/M50 and shYAP1-GFP/M50-1 as indicated. n=3.


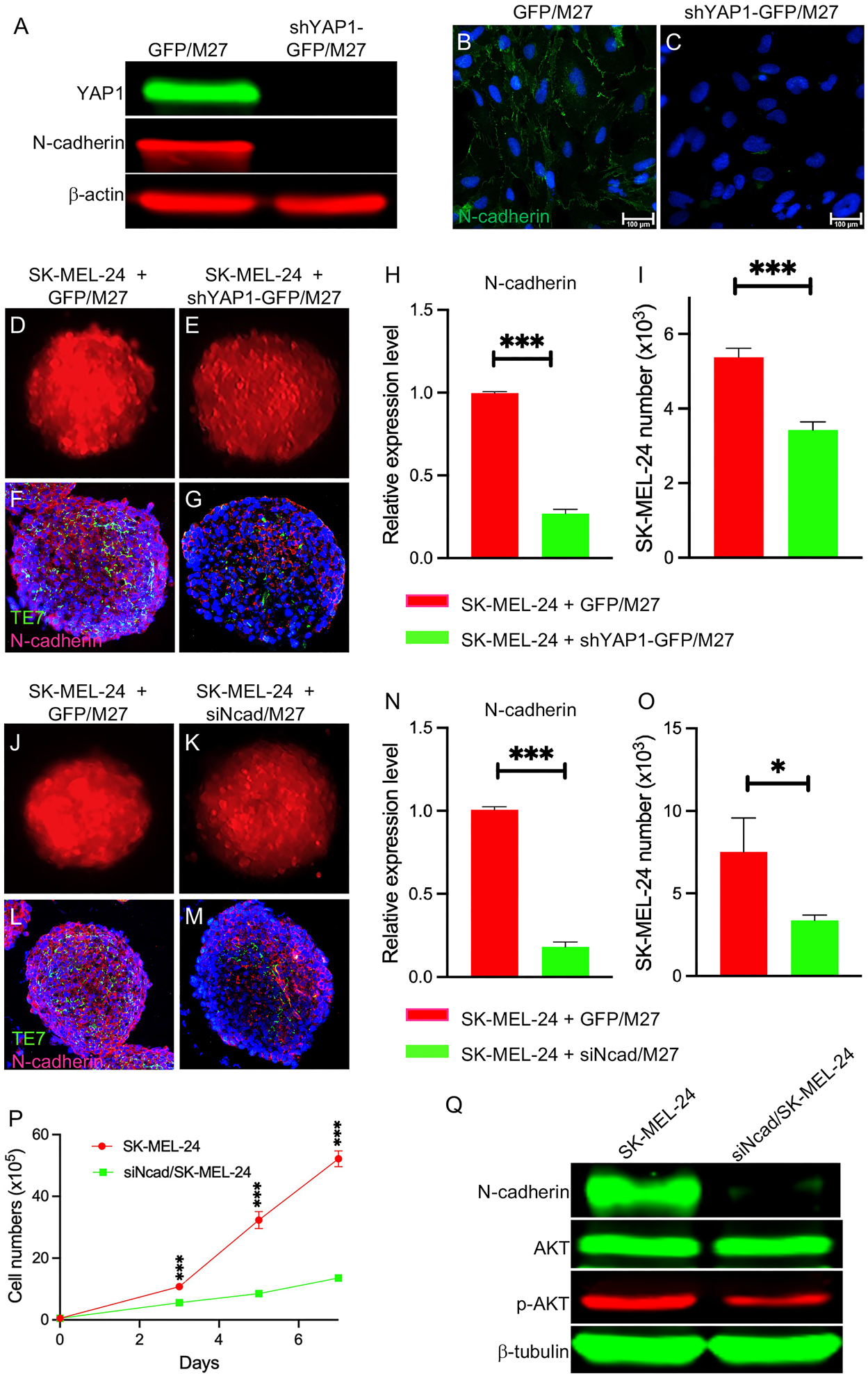


**Figure S7. N-cadherin deficiency in CAFs leads to the downregulation of N-cadherin and AKT signaling in melanoma cells.** A. Western blot shows that silencing YAP1 expression in M27 cells led to the loss of N-cadherin. B-C. Images show N-cadherin staining of GFP/M27 and shYAP1-GFP/M27 with blue DAPI nuclear counterstaining. D-E. Fluorescence images show RFP-tagged SK-MEL-24 cells in co-cultured spheroids as indicated. F-G. Images show TE7 and N-cadherin staining of co-cultured spheroids as indicated using an anti-TE7 antibody (green) and an anti-N-cadherin antibody (red). H. Quantitative analysis of N-cadherin expression in spheroids co-cultured as indicated. n=3. I. Graph shows the numbers of SK-MEL-24 cells in co-cultured spheroids as indicated. n=6. J-K. Fluorescence images show RFP-tagged SK-MEL-24 cells in co-cultured spheroids as indicated. L-M. Images show TE7 and N-cadherin staining of cocultured spheroids as indicated using an anti-TE7 antibody (green) and an anti-N-cadherin antibody (red). N: Quantitative analysis of N-cadherin expression in co-cultured spheroids as indicated. n=3. O. Graph shows the numbers of SK-MEL-24 cells in co-cultured spheroids as indicated. n=6. P. Comparison of the numbers of SK-MEL-24 cells transfected with scramble siRNA and N-cadherin siRNA (siNcad/SK-MEL-24) in culture for seven days. n=3. Q. Western blotting shows N-cadherin, AKT, and p-AKT expression in SK-MEL-24 cells transfected with scramble siRNA and siNcad/SK-MEL-24. β-Tubulin was used as a loading control. For all staining pictures, the scale bar represents 100 µm. In all statistical graphs, data are represented as the mean ± SD. *, P≤0.05; **, P≤0.01; ***, P≤0.001; ns, not significant.


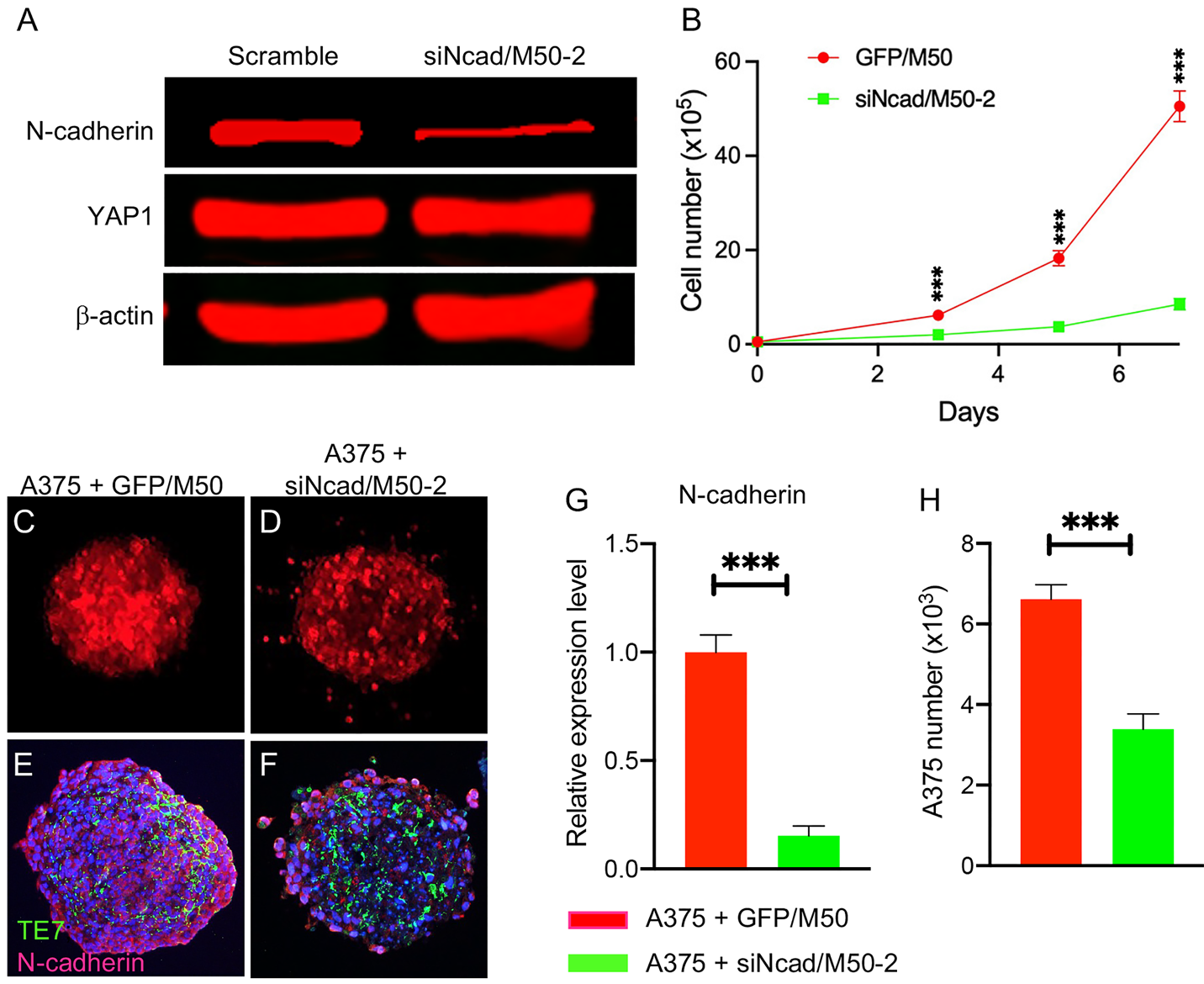


**Figure S8. N-cadherin deficiency in CAFs leads to the downregulation of N-cadherin and AKT signaling in melanoma cells.** N-cadherin expression in M50 was silenced using siRNA S2773 and named as siNcad/M50-2. A. Western blot shows that N-cadherin expression was downregulated in siNcad/M50-2. B. Comparison of the numbers of siNcad/M50-2 and GFP/M50 cells in culture for seven days. n=3. C-D. Fluorescence images show RFP-tagged A375 cells in spheroids co-cultured with siNcad/M50-2 and GFP/M50. E-F. Images show TE7 and N-cadherin staining of co-cultured spheroids as indicated using an anti-TE7 antibody (green) and an anti-N-cadherin antibody (red). G: Quantitative analysis of N-cadherin expression in spheroids co-cultured with siNcad/M50-2 and GFP/M50 by qPCR. n=3. H. Graph shows the numbers of A375 cells in cocultured spheroids as indicated. n=3. For all staining pictures, the scale bar represents 100 µm. In all statistical graphs, data are represented as the mean ± SD. *, P≤0.05; **, P≤0.01; ***, P≤0.001; ns, not significant.
